# Intravesical Onabotulinum Toxin A Injection Paradigms for Idiopathic Overactive Bladder: A Scoping Review of Clinical Outcomes, Techniques, and Implications for Practice and Future Research

**DOI:** 10.3390/toxins17050211

**Published:** 2025-04-23

**Authors:** Ekene Enemchukwu, Hodan Mohamud, Shada Sinclair, Victoria Harbour, Raveen Syan, Michael Kennelly, Susanna Gunamany

**Affiliations:** 1 Department of Urology, Stanford School of Medicine, Palo Alto, CA 94304, USA; sssinc@stanford.edu (S.S.); vharbour@stanford.edu (V.H.); susannag@stanford.edu (S.G.); 2 Temerty Faculty of Medicine, University of Toronto, Toronto, ON M5S1A1, Canada; hodan.mohamud@mail.utoronto.ca; 3 Department of Urology, Miller School of Medicine, University of Miami, Miami, FL 33136, USA; raveen.syan@med.miami.edu; 4 Department of Urology, Atrium Health—Carolinas Medical Center, Wake Forest University School of Medicine, Winston-Salem, NC 27101, USA; michael.kennelly@atriumhealth.org

**Keywords:** onabotulinumtoxinA intravesical injection, urge urinary incontinence, overactive bladder, idiopathic, injection paradigm, trigone, suburothelial

## Abstract

Introduction and Objectives: Onabotulinum toxin A (BTXA) is an effective treatment for refractory idiopathic overactive bladder (iOAB). Given the wide spectrum of patient factors and combination of symptoms, a tailored approach to management is needed. This scoping review assesses injection paradigms for iOAB. Prior studies have established the safety and efficacy of BTXA injections, and this review focuses on exploring variations in injection techniques that may inform more tailored approaches and support future research toward optimizing patient outcomes. Methods: We conducted a systematic literature search. Inclusion criteria included full-text English language and primary research studies assessing outcomes in adults undergoing BTXA for iOAB. Findings are summarized using narrative synthesis. Results: Forty-three articles were identified. Key findings include fewer injections (1–10 vs. 20–40) maintains efficacy while reducing procedure time, discomfort, and retreatment hesitancy. Durability appears to be lower with suburothelial and bladder base injections and higher with detrusor and bladder body injections, though these may carry an increased risk of urinary retention requiring clean intermittent catheterization. Trigone inclusion appears safe and effective without increased vesicoureteral reflux risk. Conclusions: Study heterogeneity and inconsistent reporting limit strong conclusions. Included injection paradigms demonstrated efficacy, high tolerability, symptom relief, and quality-of-life improvements with few adverse events. Further research is needed to refine optimal injection strategies to enhance patient comfort, maximize efficacy, and minimize adverse events. Future studies should ensure comprehensive data collection to clarify these associations.

## 1. Introduction

Overactive bladder (OAB) is a chronic, debilitating condition characterized by urinary urgency, frequency, and nocturia with or without urgency urinary incontinence (UUI) occurring in the absence of other pathology [1]. The reported prevalence of OAB varies widely, between 16.5% and 43% in the United States [2,3,4,5], and increases with age [4,6,7]. It negatively impacts health-related quality of life (QOL), productivity, sleep, sexual health, and mental well-being [8,9].

Evidence-based guideline-recommended treatments, include behavioral therapy, pelvic floor exercises, pharmacotherapy, and minimally invasive therapies. The recent 2024 American Urological Association (AUA) and Society of Urodynamics, Female Pelvic Medicine and Urogenital Reconstruction (SUFU) OAB guidelines emphasize shared decision-making to incorporate patient goals and identify the most appropriate therapy or combination of therapies for each patient [10]. This encourages a more tailored approach to OAB management by considering the patient’s specific symptoms, needs, values, and preferences. This paradigm shift highlights the need for rigorous study of tailored OAB therapies to optimize outcomes and minimize adverse effects.

### 1.1. BTXA Mechanism of Action

Our understanding of onabotulinum toxin A’s (BTXA) mechanism of action offers a unique opportunity to tailor therapy based on idiopathic OAB (iOAB) phenotype and other individual patient factors. BTXA acts on synaptic vesicle glycoprotein (SV2), present on over 90% of parasympathetic nerve fibers in the detrusor muscle and approximately half of the sensory nerve fibers in the suburothelium [11,12]. When BTXA binds SV2 at the neuromuscular junction, it inhibits the release of acetylcholine and reduces ATP and substance P release, reducing bladder contractility and the micturition reflex [8,11,12].

The normal bladder wall is 3–6 mm thick and consists of the urothelium, suburothelium, detrusor muscle, and adventitia, each supporting its function [13]. The layers of the bladder play a key role in function by integrating sensory and motor signaling. Presynaptic nerve terminals for sensory function are primarily in the suburothelium, which contains a dense network of afferent fibers mediating bladder filling and urgency. Motor function is controlled by parasympathetic efferent nerves in the detrusor muscle, where acetylcholine release activates muscarinic receptors to facilitate detrusor contraction. Understanding the precise location of these presynaptic nerve fibers is key to optimizing BTXA placement, allowing us to target sensory and motor components of bladder function. Once BTXA is internalized by a presynaptic nerve, it can only affect that specific nerve, concentrating its effects within that nerve. However, if BTXA spreads across multiple nerve terminals, therapeutic outcomes are expected to be more significant.

While trigone-sparing techniques were initially employed to minimize extravesical spread and the theoretical risk of vesicoureteral reflux (VUR), studies have not shown an increased risk of VUR with trigonal injections [14,15]. This is particularly relevant given the unique anatomy of the trigone. Unlike the bladder walls, the trigone lacks detrusor muscle fibers, but it contains a dense network of sensory fibers expressing SV2 that contribute to the sensory arm of the micturition reflex. Targeting these sensory nerves in the trigone may enhance efficacy while reducing the risk of urinary retention in high-risk iOAB populations [16]. Additionally, the number, volume, and location of injections may impact pain, tolerability, and therapy adherence rates [17].

### 1.2. Rationale for Injection Paradigms

BTXA is an effective, Food and Drug Administration (FDA)-approved therapy for the management of idiopathic OAB (iOAB) that has demonstrated clinical efficacy and safety in two pivotal phase III trials that utilized an intradetrusor, low-volume (0.5 mL), 20-injection, trigone-sparing injection paradigm [8,11]. This injection technique has been shown to significantly reduce urinary urgency and UUI episodes, with meaningful improvements in QOL and a low risk of transient urinary retention, hematuria, and urinary tract infection (UTI).

Many injection paradigms have been described, varying in injection depth (suburothelium vs. detrusor), site (trigone vs. trigone-sparing vs. bladder base vs. detrusor), number, volume, BTXA concentration, and needle size. Currently, there is no consensus on the optimal injection paradigm, and previous reviews have been limited by the exclusion of males and the inclusion of mixed populations with idiopathic overactive bladder (iOAB) and neurogenic detrusor overactivity (NDO) [18,19,20]. This scoping review provides an overview of the current literature on the efficacy of various intravesical BTXA injection paradigms for adults with iOAB, focusing on clinical outcomes and adverse events. It aims to clarify the impact of different injection parameters, identify research gaps, and propose suggestions for future investigation.

## 2. Results

**Figure 1:**
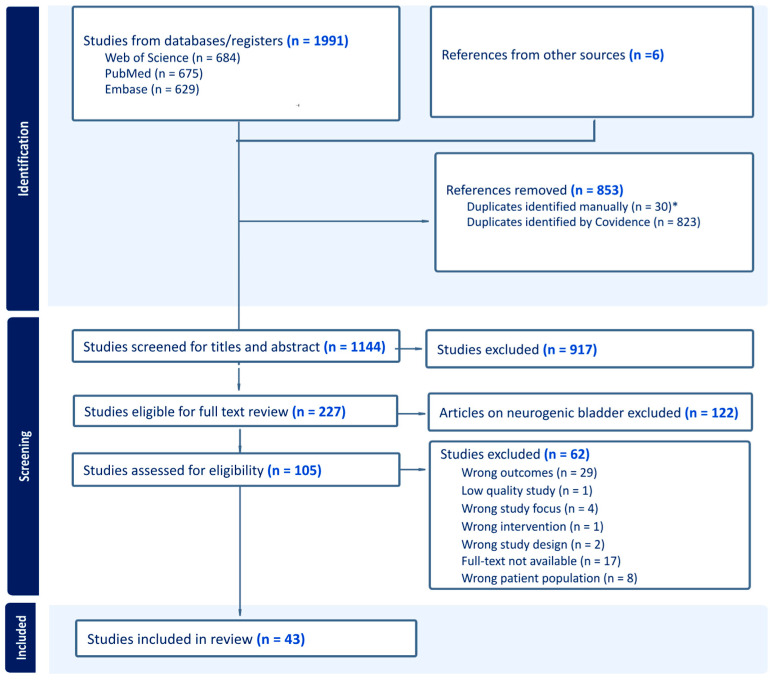
PRISMA flow diagram depicting the study selection process. * Jiang et al. (2017) [21] were excluded as duplicates due to the identical patient population, treatment, outcomes, and findings of Hsiao et al. (2016) [22].

Of 1991 records, 43 studies met the inclusion criteria (Table 1) and were included (Figure 1), representing 6740 study participants, avoiding duplication from secondary analyses. Of these, 82.4% were women, with a mean age of 62.7. Supplemental Table S1 provides an overview of the injection paradigm characteristics and outcomes. Supplemental Table S2 details the following parameters: site and depth of injection, number of injections, and volume/concentration.

Injection Protocols: The majority of included studies used a 100–200 U dose (range: 50 U–300 U) of BTXA, administered in 0.5 mL (range: 0.2–10 mL) aliquots across 20 injection sites (range: 1–40) into the detrusor wall, sparing the trigone, under local anesthesia with a rigid or flexible cystoscope and a 23-gauge needle (Supplemental Tables S1 and S2).

### 2.1. Dose Efficacy and Adverse Events

The current FDA-approved starting dose for intravesical BTXA is 100 U, which can be increased to 200 U with inadequate response [23]. This recommendation is based on a phase II randomized trial by Dmochowski et al. (2010), which identified a dose–response relationship in UUI rates with minimal improvement beyond 150 U and reduced efficacy at 50 U. Most studies (35 of 43, or 81.4%) included in this review assessed the 100 U dose of BTX alone or as a treatment arm [8,9,11,14,22,24,25,26,27,28,29,30,31,32,33,34,35,36,37,38,39,40,41,42,43,44,45,46,47,48,49,50,51,52,53,54]. Three studies assessed the impact of 50 U (3/43; 6.9%) [9,24,52], six assessed 150 U (6/43: 14%) [9,24,26,38,51,52], fourteen assessed 200 U (14/43: 32.5%) [9,15,24,25,29,39,40,44,45,52,55,56,57,58], and six included doses exceeding 200 U (4/43; 9.3%) [9,24,29,38,52,56] (Supplemental Table S1).

**50 U Dose:** In a phase II placebo-controlled, randomized, dose-ranging trial (*n* = 313) [24,52], changes in UUI episodes/week and urodynamic parameters were assessed in placebo, 50 U, 100 U, 150 U, 200 U, and 300 U groups. The 50 U dose outperformed the placebo in UUI resolution (15.9% vs. 29.8%), but demonstrated both reduced and less sustained efficacy outcomes compared to the 100 U or higher doses (37–57%). However, the 50 U dose exhibited the lowest rates of adverse events, including urinary retention with the need for clean intermittent catheterization (CIC) (5.4% vs. 10.9–12.2%). (Supplemental Table S1) Patient-reported outcomes highlight this limited efficacy [9], with no significant benefit in health-related QOL improvements when compared to placebo, while higher doses (>100 U) demonstrated significant improvements [9]. Overall, the 50 U dose reduced adverse events, but provided poorly sustained, limited urodynamic efficacy and HRQOL improvements.

**100 U Dose:** The dose–response relationship observed in the phase II trials identified 100 U as the optimal balance of symptom improvement and adverse events [9,24,52]. The 100 U dose offered superior efficacy in UUI resolution compared to 50 U (50 U: 29.8% vs. 100 U: 37%), with minimal additional benefit observed at the 150 U dose (40.8%). Similarly, adverse events, such as elevated PVR requiring CIC, were dose-dependent, but less frequent at 100 U (10.9%) compared to higher doses (150 U: 20%, 200 U: 21.2%, 300 U: 16.4%) [52]. Phase III and IV trials confirmed the safety and efficacy of 100 U, with high UUI resolution rates (28.9–32%) and low CIC rates (6.1–6.9%) [8,11,12].

**150 U Dose:** In a small RCT (*n* = 42), Cohen et al. (2009) observed no differences between 100 U and 150 U doses in clinical outcomes, including dry rates, urodynamic parameters, QOL, urinary retention requiring CIC (*n* = 2, 1 in each group), or UTI [38]. Similarly, in a 3.5-year extension of the phase II and III BTXA trials, Nitti et al. (2016) (*n* = 829) observed equivalent outcomes in the 286 patients who received 150 U and the remainder of the cohort who received 100 U [26]. These studies support prior findings that 150 U offers no additional benefit over 100 U, demonstrating similar improvements in OAB symptoms, urodynamic parameters, and QOL, but with a higher risk of CIC.

**200 U Dose:** In an RCT, Amundsen et al. (2016) compared BTX-A, 200 U, and sacral neuromodulation (SNM) for refractory UUI in women [57]. At 6 months, BTX-A showed a significant reduction in UUI episodes (−3.9 vs. −3.25, *p* < 0.01) and higher UUI resolution rates (20% vs. 4%, *p* < 0.001) vs. SNM. Both groups reported similar patient-perceived improvement (71% vs. 68%, *p* = 0.82). Adverse events were more frequent with BTX-A, including UTI (35% vs. 11%, *p* < 0.001) and elevated PVR requiring CIC (2.1% vs. 0%, *p* = 0.04). Other studies also demonstrated significant reductions in UUI at the 200 U dose vs. placebo (−57.5 vs. 9.3%) (*p* < 0.01) [56], with low (4.5%) CIC rates. CIC rates (18–21.2%) vary across studies, often depending on how urinary retention requiring CIC was defined [39,52].

**Special Populations:** Older Adults: BTXA 100 U is effective in older adults. In a retrospective study (*n* = 192), Ou et al. (2023) reported similar subjective cure rates: 66.9% in younger (<75 years) and 60% in older (≥75 years) patients with iOAB. Adverse event rates, including urinary retention (8.7% vs. 12.3%, *p* = 0.42) and UTI (14.2% vs. 9.2%, *p* = 0.33), were comparable between groups [41].

**Summary:** The 100 U dose of BTXA is optimal, with minimal additional improvement at 150 U and reduced efficacy at 50 U. Higher doses, such as 200 U, offer higher symptom relief, but carry a higher risk of CIC, while 100 U appears to be equally effective in both “younger” and “older” adults. Further studies are needed to determine which iOAB population may benefit from the alternative doses (e.g., 50 U dose) to reduce AE risk.

### 2.2. Site and Depth of Injection

#### 2.2.1. Outcomes Based on the Depth of Injection (Suburothelial vs. Detrusor)

**Figure 2:**
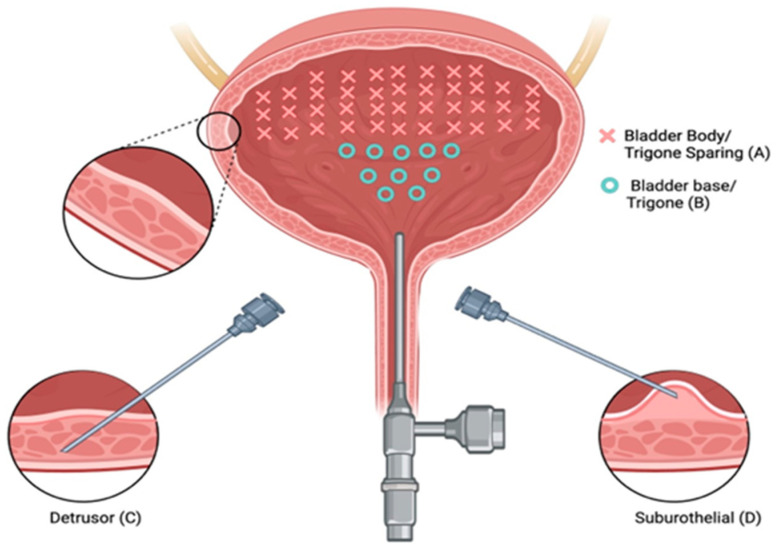
Three types of intravesical botulinum toxin A injections. Intravesical injections at 40 sites with trigone sparing (A) using suburothelial injection (D) and/or detrusor injection (C). Bladder base and trigonal injections (B) were performed at 10 sites.

**Figure 3:**
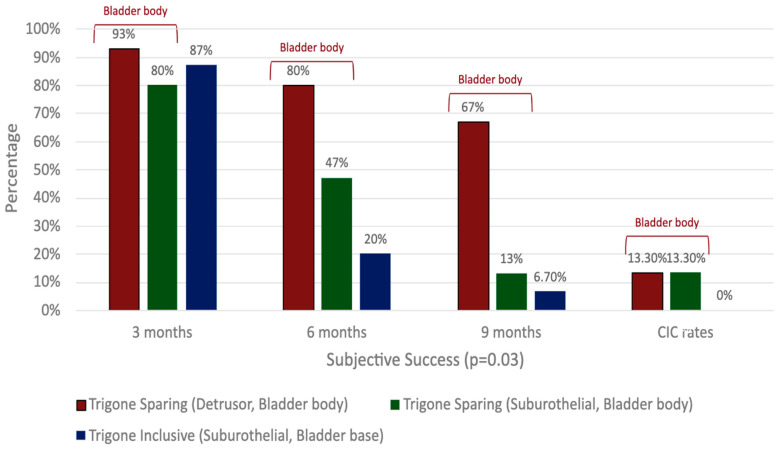
Comparison of clinical outcomes in detrusor, suburothelial, and bladder base injections.

There is a paucity of studies evaluating the impact of injection depth on treatment outcomes, with only two studies meeting the inclusion criteria for this review. In an RCT of refractory iOAB (*n* = 45), Kuo (2007) compared three injection techniques using 100 U BTXA: trigone-sparing detrusor, trigone-sparing suburothelial, and trigone-including bladder base suburothelial injections [14] (Figure 2). At 3 months, there were no statistically significant differences in efficacy (*p* = 0.07) or QOL improvement (*p* = 0.56) between groups (Figure 3). However, detrusor injections were more durable than suburothelial bladder body injections, followed by suburothelial bladder base injections (Figure 3). Urinary retention requiring CIC occurred more frequently in the detrusor (*n* = 2, 13.3%) and suburothelial bladder body (*n* = 2, 13.3%) groups than the suburothelial bladder base group (0%). No VUR was observed. In a subsequent study (*n* = 105), Kuo (2011) compared trigone-sparing detrusor, bladder body/trigone, and bladder base/trigone injections, finding no significant difference in success rates at 3, 6, or 9 months. At 12 months, the bladder base/trigone group had numerically lower success rates (37%) than the bladder body (49%) and bladder body/trigone (50%) groups, though these differences were not statistically significant. CIC rates were 0% for bladder base, 5% for bladder body, and 11% for bladder body/trigone, though the study was underpowered [53].

Overall, BTXA demonstrates efficacy independently of the injection depth [31,33,51]. Suburothelial and bladder base injections may be less durable but have lower CIC rates than detrusor or bladder body injections. Bladder base/trigone injections may also be less durable than bladder body injections, regardless of trigone involvement, but with the benefit of lower CIC rates.

**Summary:** Detrusor injections are likely more durable than suburothelial injections, though they were associated with higher rates of urinary retention requiring CIC. In contrast, suburothelial injections improved symptoms with a lower risk of CIC.

#### 2.2.2. Trigone-Sparing vs. Trigone-Including Injection Paradigms

Most studies in this review focused on the impact of trigone-sparing injection techniques, with only eight (8/43; 18.6%) examining trigone-including injections in patients with iOAB [14,15,32,35,37,48,53,57].

#### 2.2.3. Trigone-Sparing Detrusor Injections vs. Trigone-Including Detrusor Injections

El-Hefnawy et al. (2021) conducted an RCT (*n* = 103) comparing trigone-sparing (*n* = 52) and trigone-including (*n* = 51) injections, administering 100 U BTXA in 20 (0.5 mL) sites via a 7-gauge needle [37]. Both groups demonstrated significant reductions in OABSS scores, with UI episodes reduced by 93% in the trigone-sparing group and 85% in the trigone-inclusive group (*p* = 0.38). Adverse events were more common in the trigone-inclusive group, with 19.6% reporting voiding difficulties and 3.9% requiring CIC for PVR >200 mL, but there were no differences in UTI rates or VUR between groups [37].

In a retrospective cohort study (*n* = 45), Ton et al. compared 100 U BTXA administered as a single 10 mL trigone-only injection (*n* = 19) vs. 20 trigone-sparing 0.5 mL injections (*n* = 26) [48]. Both groups had similar durability and adverse event rates, but the trigone-only group had a lower PVR (113 mL vs. 160 mL, *p* < 0.02), a lower CIC rate (5.3% vs. 17.4%, *p* < 0.02), and shorter procedure times (4.3 min vs. 5.7 min, *p* < 0.01). No VUR was observed. In an RCT, Kuo (2007) (see Section 2.2.1) observed superior durability in outcomes in the trigone-sparing bladder body (detrusor and suburothelial) group compared to the trigone-inclusive bladder base group (*p* = 0.01, Figure 3, Supplemental Table S2). However, CIC rates were lower in the trigone-inclusive group (0% vs. 13.3%), with no VUR observed [14].

#### 2.2.4. Trigone-Inclusive Detrusor Injections vs. Placebo

MacDiarmid et al. (2023) explored an alternative injection paradigm using ten injections (1.0 mL), eight administered to the posterior wall and two to the bladder trigone [35]. In this phase IV multicenter, randomized, double-blind, placebo-controlled trial (*n* = 120), the BTXA group demonstrated significantly greater reductions in daily UI episodes at week 12 (−2.9) vs. placebo (−0.3) (*p* < 0.0001). Trigonal injections were generally well tolerated, with low adverse events rates in both groups (UTI: 15.4% vs. 5.1%, CIC: 2.6% vs. 0%, dysuria: 5.1% vs. 2.6%, in BTXA vs. placebo, respectively). VUR was not reported [35].

#### 2.2.5. Trigone-Inclusive Injections vs. No Comparator

Onem et al. (2018) conducted a multicenter, prospective single-arm study (*n* = 80) to investigate the efficacy and safety of administering 100 U BTXA in 20 injections (0.5 mL), including the trigone [32]. At 3 months, bladder diary parameters (UUI, urgency, and urinary frequency) and urinary incontinence QOL (I-QOL) scores showed significant improvements from baseline (*p* < 0.05). The treatment satisfaction rate was 82.5%. Adverse events included uncomplicated UTI (6.25%) and hematuria (6.25%), and CIC was initiated in three patients (3.8%). VUR was not reported.

**Summary:** Trigone-inclusive protocols demonstrate good efficacy, no VUR, and low adverse event rates. One study suggests that patients are more likely to experience subjective voiding difficulties with trigone inclusion, while two others indicate that retention rates are lower when the trigone is included. However, the heterogeneity in trigone injection techniques across studies (e.g., trigone only, trigone with bladder base, or trigone with detrusor) limits the ability to draw meaningful differences in adverse events.

### 2.3. Number of Injections

Studies have explored pain reduction strategies for the administration of BTXA, including minimizing injection sites [17]. Thirty-eight studies (90.5%) reported the number of BTXA injections administered. Most (*n* = 22/43; 51.2%) used a standard 20-injection technique [8,9,11,14,22,25,26,27,28,29,30,31,32,34,36,37,38,42,45,52,56,57], which consistently improved outcomes (frequency, nocturia, urgency, dry rates, and QOL) compared to baseline or placebo [8,9,22,25,26,27,28,29,30,31,32,34,36,37,38,42,45,52,53,56,57]. Seven studies reported significant improvements in QOL [8,9,12,26,27,32,40]. Alternative injection regimens (e.g., 10, 30, 40 injections) were also evaluated, with some directly comparing injection paradigms (e.g., 5 vs. 20 injection sites) [42,43,47]. Pain scores were assessed in only three studies [42,47,49].

#### 2.3.1. 10 vs. 40 Injections

In an RCT (*n* = 45), Kuo (2007) (see Section 2.2.1) compared 100 U BTXA administered via 40 detrusor, 40 suburothelial, and 10 suburothelial bladder base injections [14]. The 40-injection groups had more durable success rates, but also experienced higher CIC rates compared to the 10-injection group (*n* = 4/30; 13.3% vs. 0%). However, since injection sites varied across groups, the relative impact of injection number vs. location remains unclear. Pain scores and procedure duration were not assessed.

#### 2.3.2. 10 vs. 20 vs. 40 Injections

In an RCT, Liao et al. (2016) compared 10, 20, and 40 suburothelial BTXA injections (100 U dose) [43]. The 10- and 40-injection groups demonstrated significant reductions in UUI episodes at 6 months, with decreases of 7.24 episodes/day (8.95 ± 10.74 to 1.71 ± 2.34) and 4.53 episodes/day (9.86 ± 13.11 to 5.33 ± 12.17), respectively, while the 20-injection group showed no significant change (decrease of 0.15 from 5.29 ± 7.64 to 5.44 ± 16.66). UTI rates were higher in the 20-injection group (31.8%) compared to the 10 (12.5%) and 40-injection groups (9.5%), with no significant differences in other adverse events. Pain scores or duration of procedures were not assessed.

#### 2.3.3. 5 vs. 20 Injection Sites (2 Studies)

DiCarlo-Meacham et al. (2023) conducted a randomized noninferiority trial *(n =* 77) comparing 100 U BTXA given in 5 “reduced” injections (2 mL/site) vs. 20 “standard” injections (0.5 mL/site) [42]. There was a significant improvement in both arms on validated questionnaires (*p* < 0.001) and overall treatment success of 68% with no statistically significant difference between groups. Urinary retention requiring CIC was observed in the 5-injection group (*n* = 2, 4.9%), but not in the 20-injection group. There was no difference in UTI rates (*p* = 0.28). Both groups had similar pain scores, but the “reduced” 5-injection group was more willing to repeat the procedure (odds ratio = 3.8, 95% CI: 1.42–10.67, *p* = 0.004).

Chang et al. conducted an RCT (*n* = 60) comparing pain and procedure time in patients with iOAB receiving 100 units of BTXA in 5 injections vs. 20 injections [47]. Patients rated their pain on a 10-point visual analogue scale (VAS), and procedure time was recorded. Results showed no significant difference in pain scores between the groups (*p* = 0.27), but the 5-injection group had a significantly shorter procedure time (76 s vs. 176 s, *p* < 0.001). There were no differences in subjective efficacy between the 5- and 20-injection groups (+2 vs. +2 on the Global Response Assessment (GRA) score) or adverse events between groups (UTI: 20% vs. 16.7% and CIC: 10% vs. 6.7%), respectively [47]. VAS scores were also similar between treatment-naïve and repeat-treatment patients (*p* = 0.27).

#### 2.3.4. 10 vs. 20 Injection Sites

In a single-blinded randomized trial, Zdroik et al. evaluated the effect of BTXA injection number and volume (100 U delivered as ten 1 mL injections vs. twenty 0.5 mL injections) on procedural pain using an 11-point numerical pain rating scale (NPRS) immediately after the procedure [49]. Local anesthesia was administered pre-procedurally via bupivacaine and NaHCO_3_ bladder instillation. The median pain scores were reported as 4 (interquartile range: 1.5–5) for the 10-injection group and 3 (interquartile range: 1–4) for the 20-injection group, with no statistically significant difference observed between the two groups (*p* = 0.82) [49]. There were no significant differences in efficacy (success rates: 46.7% vs. 40%, *p* = 0.8) or safety (UTI: 2/19 (10.5%) vs. 2/21 (9.5%), CIC: 1/21 (4.8%) vs. 2/19 (10.5%) in the 10- and 20-injection group, respectively).

#### 2.3.5. 1 Injections vs. 20 Injections

In a retrospective cohort study, Ton et al. (see Section 2.2.3) assessed differences in outcomes for BTXA 100 U given in a single trigonal injection vs. 20 trigone-sparing injections [48]. The authors observed no significant differences in inter-injection intervals or UTI rates between groups. However, the single-injection (trigone only) group had lower rates of urinary retention requiring catheterization (5.3% vs. 17.4%, (*p* = 0.014)). Procedure time was lower in the single-injection group (4.3 ± 2.02, 5.7 ± 2.9, *p* = 0.003). Differences in pain scores were not assessed or reported [48].

#### 2.3.6. 30 Injections

In a prospective cohort study, Okamura et al. (2013) evaluated (*n* = 17) participants with idiopathic OAB who were treated with 100 U BTXA reconstituted in 15 mL of 0.9% normal saline and injected 0.5 mL for a total of 30 submucosal injections. They observed a significant reduction in urinary frequency, urgency, and urgency urinary incontinence, with no adverse events reported [33]. No episodes of urinary retention or catheterization were observed. Pain was not assessed.

**Summary:** While injection number alone does not appear to impact efficacy, it is important to consider BTXA’s mechanism of action, as other factors such as volume, distribution, depth, and location all contribute to efficacy. Given that injection volume and number are interconnected, caution is warranted in attributing efficacy solely to the number of injections. One study found that five injections were associated with a greater willingness to repeat the procedure, even though pain scores were similar for higher numbers of injections.

### 2.4. Dilution and Volume

#### 2.4.1. Dilution

Among the 43 studies, 34 (79%) reported data on the volume and dilution of BTXA injections, of which 13 administered 0.5 mL per injection with 10 U/mL concentrations. Studies employing this volume and concentration demonstrated significant improvements in OAB symptoms, including reductions in urgency, micturition episodes, nocturia, and improved QOL scores compared to baseline or placebo groups [8,11,12,14,25,26,27,37,41,42,43,44,52]. Adverse events commonly reported in these studies included UTIs, dysuria, bacteriuria, urinary retention, hematuria, and the need for CIC.

One study with an alternative BTXA dilution strategy met the inclusion criteria for this review (see Section 2.3.6). In a prospective cohort study with no comparison group, Okamura et al. (2013) evaluated (*n* = 17) participants treated with 100 U BTXA reconstituted in 15 mL (6.6 U/mL) and injected 0.5 mL (3.3 U/injection) for a total of 30 submucosal injections. They observed a significant reduction in urinary frequency, urgency, and urgency urinary incontinence, with no urinary retention reported [33].

#### 2.4.2. Volume

##### 0.5 mL vs. 2 mL Injection Volume

Chang et al. (see Section 2.3.3) compared the 100 U BTXA dose (*n* = 40) administered as either 2 mL (5 injections) or 0.5 mL (20 injections) aliquots. Pain scores were similar between groups (*p* = 0.27), but procedure time was significantly shorter with higher volume injections (fewer injections) (76 s vs. 176 s; *p* < 0.001). No differences in GRA score (efficacy) or adverse events were observed [47].

##### 0.5 mL vs. 1 mL Injection Volume

In a single-blinded randomized trial, Zdroik et al. (see Section 2.3.4) compared the outcomes of 100 units of BTXA administered in 1 mL (10 injections) vs. 0.5 mL (20 injections) aliquots. There was no difference in efficacy (*p* = 0.82), adverse events, or NPRS scores immediately after the procedure (*p* = 0.84) [49].

##### 0.5 mL vs. 10 mL Injection Volume

In a single-institution, retrospective chart review, Ton et al. (see Section 2.2.3) compared outcomes for 100 U BTXA administered in a single 10 mL injection into the trigone vs. 0.5 mL (20 injections) in a trigone-sparing injection pattern. However, in the trigone-only group, the needle was tunneled intramurally towards the dome, and methylene blue was used for visualization and observation of spread in both groups, though the degree of spread was not measured. The procedure setting or method of anesthesia was not reported. While there were no differences in GRA scores, the high-volume (10 mL), single-injection group had significantly shorter procedure time (4.3 min vs. 5.7 min, *p* < 0.01). However, anesthetic details and pain scores were not reported. In addition, the differences observed in PVR outcomes were not clinically significant (113 ± 111(0–500) vs. 160 ± 134(0–850); however, it is unclear if these observed differences were the result of the location (trigone) or localization (volume/number) of the injection [48].

**Summary:** There are no head-to-head studies comparing different injection volumes with the same dose and number of injections, but BTXA has proven effective at various volumes and concentrations. While using higher volumes with fewer injections reduces procedure time, the 1.4 min difference may not be clinically significant and should be weighed against patient tolerability, which was not formally assessed or reported.

### 2.5. Needle Size

Needle size has traditionally been discussed in the context of safety. In more contemporary literature, needle size has been explored as a potential contributor to pain and tolerability of BTXA therapy under local anesthesia [17].

There is limited literature available assessing the impact of injection needle size on intradetrusor BTXA outcomes and adverse events. Current recommendations are for an ultrafine needle (22 to 27 gauge and 4 mm long) to mitigate the risk of bladder wall perforation or inadvertent extravesical administration of BTXA [19,59,60]. The normal bladder wall is 4–6 mm, and consequently most commercially available needles are 2–5 mm in length. Retractable needles are available to help compress and tamponade any areas of significant bleeding.

However, few studies report needle size or length, and none of the studies included in this review directly compared needle sizes, with only 8 out of 42 studies (19%) specifying the injection needle used [14,28,36,37,38,43,45,56]. Various sizes were reported, including 7 gauge by El Hefnawy et al., performed under sedation (*n* = 1) [37]. Other sizes included 18-gauge (*n* = 1), 22-gauge (*n* = 2), 23-gauge (*n* = 3), and 27-gauge (*n* = 1) needles. Despite the variance in needle size, all eight studies reported a significant improvement in clinical and QOL endpoints compared to baseline or placebo groups. Notably, there were no discernible differences in adverse events, such as hematuria or UTI, relative to injection needle size. Pain scores were not routinely reported in these studies.

**Summary:** Needles come in several sizes and lengths, flexible vs. rigid and retractable vs. fixed. Overall, needle size does not appear to directly impact efficacy or safety, though the studies available are too few in number to draw any meaningful conclusions on the impact of needle size on efficacy outcomes.

### 2.6. Scope Type

In the literature, flexible cystoscopy is reported to be more comfortable and generally preferred [61,62] compared to rigid cystoscopy for both men [63,64] and women [65] of all age groups. Other studies report negligible differences in pain perception between the two methods, suggesting that differences in tolerance may be minimal [66,67,68]. However, rigid scopes are generally easier to use, have a lower learning curve, and offer better control, improved visualization, and greater precision. They are also typically less expensive and have lower maintenance costs. All studies included in this review used rigid, flexible, or a combination of both cystoscope types. Recorded cystoscope sizes ranged from 14 to 23 French. None of the included studies investigated the effect of scope type (rigid vs. flexible) or size on ease of therapy administration, therapy outcomes, or patient tolerability (e.g., pain scores). However, several studies utilizing only rigid (*n* = 6) cystoscopes [14,27,40,41,43] or only flexible (*n* = 3) cystoscopes [25,36,38] reported high therapeutic success and low adverse event rates.

**Summary:** The tolerability of a flexible vs. rigid cystoscopy during intravesical BTXA administration has not been directly studied, though both types are associated with good efficacy and low adverse rates.

## 3. Discussion

The variation in published BTXA injection techniques makes it difficult to effectively evaluate the impact of individual parameters. This challenge is further compounded by the heterogeneity of iOAB, which includes OAB-wet, characterized by UUI, and OAB-dry, defined by urgency and frequency without incontinence. Study outcomes also vary, with some focusing on UUI, urgency, or frequency and others on QOL parameters, making it difficult to compare efficacy and treatment outcomes across studies. These factors make it necessary to establish standardized injection protocols and consistent patient outcome reporting to allow for meaningful comparisons across studies.

Early dose-escalation studies established the dose–response relationship for reductions in UUI, leading to FDA approval of 100–200 U for iOAB due to its optimal balance of efficacy and safety [52]. While urinary retention, CIC, and UTI rates increase with higher doses, the benefits of dose escalation plateau above 200 U. In comparison to the 100 U dose, lower doses (50 U) reduce adverse events, but lack efficacy, whereas doses of 150 U provide marginal gains with a greater risk of complications. However, in select patients at higher risk of urinary retention, incremental dose adjustments may help optimize the balance between efficacy and adverse events. Dose selection should be individualized, considering patient-specific factors such as baseline post-void residual volume, risk of urinary retention, and tolerance for CIC. Further studies are needed to assess the role of lower doses (e.g., 50 U) in select patients at increased risk of urinary retention (e.g., males, frail, older adults, low Qmax and bladder contractility index [69], and to determine baseline factors (e.g., urodynamic parameters) that may predict success with the 50 U dose.

Similarly, trigone-sparing injections may offer potential benefits for iOAB patients at increased risk of retention. Studies comparing trigone-sparing and trigone-inclusive approaches suggest that including the trigone is safe and does not increase the risk of VUR. While trigone-including injections are effective, the addition of detrusor injections may increase the risk of voiding difficulty due to BTXA’s impact on both motor and sensory neural pathways. In one study, the authors reported higher rates of subjective voiding difficulty and CIC with trigone-including detrusor injections compared to trigone-sparing detrusor injections [37]. However, CIC rates remained low (3.9%), and this difference was not observed in other studies included in our review. In contrast, trigone-inclusive bladder base injections without detrusor injections may result in lower rates of urinary retention requiring CIC compared to trigone-sparing detrusor injections, but they may be less durable [14,53]. This suggests that the trigone itself is not the critical factor, but rather that the distribution of motor neurons (e.g., detrusor vs. bladder base injections) is more important. Further adequately powered studies are needed to determine which iOAB patient populations would benefit most from detrusor, bladder base, or combination injection approaches.

Published studies use various combinations of injection depths, sites, numbers, and volumes, limiting our ability to draw clear conclusions on outcomes and adverse events. Individually, these variations do not appear to impact efficacy or adverse event rates. While the 20-injection regimen was most commonly used, alternative paradigms (1, 5, 10, 30, 40 injections) show similar efficacy and adverse events. One study (Liao 2016) found reduced efficacy with the 20-injection regimen compared to the 10- and 40-injection paradigms; however, the 20-injection group had less severe UUI at baseline [43]. In two mixed IDO and NDO cohort studies that did not meet the criteria for the current review, the authors found that fewer injections (1–4) had similar efficacy and duration of effect compared to 30 injection sites [70,71]. Overall, the literature on iOAB patients suggests that reducing the number of injections (1–10 injection) reduces procedure time, improving patient comfort and increasing willingness to repeat therapy [47,48]. Further adequately powered studies are needed to confirm these findings.

While the variations in injection numbers do not appear to impact efficacy or adverse event rates, further exploration into alternative injection approaches is warranted. One such approach, submucosal injections, may offer a promising alternative for tailored therapy in iOAB-dry patients by targeting sensory nerve activity while minimizing impact on detrusor contractility, as some residual BTXA likely diffuses across layers. A study by Kuo et al. suggests that suburothelial injections may be less durable than detrusor injections [53]. These findings suggest that injection depth may influence treatment efficacy, with detrusor injections showing greater durability compared to suburothelial injections. Adverse events, such as urinary retention, were more common in the detrusor and bladder body groups (13.3%, *n =* 2/15) than in the trigone-inclusive bladder base group (0%, *n =* 0/15), suggesting that the injection site may be more important than injection depth for urinary retention outcomes. In summary, injection depth (detrusor > suburothelial) appears to influence treatment durability, while injection site (bladder walls > base) appears more closely associated with urinary retention. However, this conclusion is based on two studies, and thus adequately powered studies are needed to clarify the impact of injection depth on both efficacy and urinary retention.

While injection depth and site may impact durability and retention rates, volume and distribution of injections (local vs. diffuse) are also critical for the spread of BTXA to presynaptic nerve terminals, located in the suburothelium and within the detrusor muscle. However, data on the impact of injection volume on efficacy and adverse events are limited due to the heterogeneity in injection site (e.g., trigone-only vs. non-trigone sparing approaches) and other interconnected parameters such as number of injections. The impact of volume on pain scores and tolerability remains underexplored in iOAB. Ton et al. evaluated 0.5 mL vs. 10 mL injection volumes, but did not assess pain or tolerability [48]. Similarly, there are limited data on how needle size affects BTXA outcomes or tolerability. The available literature suggests that ultrafine needles improve patient safety and comfort. A mixed IDO and NDO cohort study, which did not meet our scoping review inclusion criteria, utilized three needle sizes (22–27 gauge, 4–5 mm length) based on supply chain availability. VAS scores showed that the 27-gauge needle caused significantly less pain compared to larger needles [31]. Additionally, factors such as the number of injections (the first three were less painful than subsequent injections) and injection location (the posterior and right bladder wall were the most painful) also contributed to pain levels. One study investigated intravesical instillation of a BTXA + hydrogel admixture to see if injection can be avoided; however, there was no improvement over placebo [72]. No studies have directly compared BTXA tolerability, ease of administration, or efficacy between rigid and flexible scopes. While flexible scopes are assumed to be more comfortable [61,62], rigid scopes generally offer better control, are easier to use, and are generally lower in cost and maintenance.

Limitations of this scoping review include heterogeneity in BTXA injection paradigms, vague or inadequate reporting of study details, underpowered studies, heterogeneous patient populations (e.g., mixed cohorts of OAB-wet and OAB-dry), and non-standardized outcome measures. The variability in outcome definitions and measurement tools across included studies, particularly for QOL assessments, may affect the comparability of findings. However, this review offers potentially practice-altering insights, particularly regarding the impact of fewer injections and improved tolerability. Previously published recommendations for injection paradigms based on a combination of patient factors, available literature, and expert opinion may provide hypotheses to guide future studies (Table 2) [73]. The exclusion of non-English literature is also a limitation of this study, as it may have omitted relevant studies from the review.

## 4. Conclusions and Future Directions

Despite substantial evidence on dose–response relationships for UUI in managing refractory iOAB, significant gaps remain in individualizing therapy. Patient-specific factors, such as age, gender, comorbidities, urodynamic parameters, baseline bladder function, OAB subtype, and treatment history (e.g., BTXA-naïve patients), are insufficiently studied. Addressing these factors is essential for objectively assessing quality and outcomes in BTXA injection paradigms, ultimately informing clinical practice and patient counseling. The literature would benefit from future RCTs that assess the comparative impact of the number, volume, and site of Botox injections on treatment efficacy, tolerability, and the incidence of adverse events. Further research is needed to identify subgroups that may benefit from tailored doses, injection numbers, and scope types while balancing treatment effectiveness, adverse events, and tolerability. Establishing a standardized minimum dataset for this research (Table 3) would be beneficial. Similarly, future trials should focus on randomized controlled designs to assess individualized dosing strategies considering patient-specific factors. Exploring the economic impact of individualized therapy could yield health-care savings, further supporting the optimization of treatment strategies. Additionally, establishing real-world data registries to track and evaluate long-term patient outcomes could provide valuable insights into the effectiveness and tolerability of botulinum toxin treatments across diverse populations.

## 5. Methodology

Given the heterogeneity of the available data, scoping review methodology was utilized. The standard methodology outlined in the Preferred Reporting Items for Systematic Reviews and Meta-Analyses (PRISMA) extension for Scoping Review checklist guidelines [74] was followed. The aim was to gain an overview of the current state of knowledge. We included studies that focused on idiopathic OAB patients receiving BTXA injection interventions without a comparison group, placebo, or alternative injection paradigm and reported efficacy and safety outcomes. This scoping review is not registered in PROSPERO, as scoping reviews are not currently accepted for registration under their criteria.

### 5.1. Study Selection

Inclusion Criteria: Our inclusion criteria were designed to encompass a broad range of studies relevant to the use of botulinum toxin, specifically BTXA, in treating idiopathic OAB in adult (>18 years) populations. We included studies involving idiopathic OAB and considered participants of both sexes without geographical restrictions. As this review was primarily focused on exploring the efficacy and safety of botulinum toxin, no specific comparison or control groups were mandated for inclusion. All papers published up until February 2024 were included (Table 1).

Exclusion Criteria: To ensure a comprehensive overview of the available literature, we considered all study designs except for systematic reviews, meta-analyses, scoping reviews, literature reviews, conference abstracts, case studies, and editorials. These criteria were established to prioritize primary research studies that provided substantive data relevant to the objectives of this review. Non-English language studies were excluded, as were those specifically addressing neurogenic detrusor overactivity. Furthermore, studies investigating botulinum toxin types other than BTXA were also excluded. Articles with no full-text accessibility were also excluded from the review.

### 5.2. Outcomes

The outcomes of interest included efficacy, including success rate, dry rate, and quality-of-life outcomes, and adverse events such as urinary tract infections, urinary retention, clean intermittent catheterization, and hematuria.

### 5.3. Search Strategy

Electronic databases, including PubMed, Embase, and Web of Science, were systematically searched by a trained librarian (AW) to identify relevant studies. Two reviewers manually removed 122 papers on neurogenic bladder during the full-text review.

A manual search of reference lists and relevant journals was conducted to supplement the electronic searches. Search terms were selected to encompass the breadth of relevant literature. They included phrases such as “overactive bladder”, “idiopathic overactive bladder”, “botulinum toxins”, “Clostridium Botulinum”, “Botulinum Neurotoxin”, “Botox”, and “OnabotulinumtoxinA”. The Boolean operators AND, NOT, and OR were used to combine the search terms effectively.

### 5.4. Data Collection and Analysis

Four reviewers were engaged in the review process and independently assessed titles and abstracts using the Covidence platform. Full-text articles were then obtained and independently assessed for inclusion by two independent reviewers, and discrepancies were resolved through discussion with a third reviewer. Data extraction was conducted using Covidence to ensure systematic and organized collection of relevant information. A narrative synthesis approach was employed to summarize findings and identify key themes across the included studies.

## Figures and Tables

**Table 1:** Inclusion and Exclusion Criteria.

Inclusion Criteria	Exclusion Criteria
Human Subjects, Adults (>18 years), Male and Female	Non-English language
Idiopathic Overactive Bladder	Neurogenic bladder
OnabotulinumtoxinA	Other types of botulinum toxins
Primary research articles	Case studies, Systematic reviews, Meta-analysis, Scoping reviews, Abstracts, Conference Abstracts, Case studies and Editorials
Outcomes: Efficacy, Adverse Events	Full text is not available or retrievable

**Table 2:** Proposed Botulinum Toxin Injection Paradigm Based on Patient Factors (Courtesy of Dr. Michael Kennelly, based on an assimilation of the literature and clinical practice on the use of BTXA over 20 years in the bladder for iOAB and Neurogenic Detrusor Overactivity).

	OAB Wet *	OAB Wet	OAB Wet	OAB Dry	NDO w CIC	NDO w/o CIC
BTX-A Naïve?	Yes	Yes	No	N/A	N/A	N/A
Dose (Units)	100	100	100–200	100	200–300	100
Location Detrusor vs. Submucosal	Detrusor	Detrusor	Detrusor	Submucosal	Submucosal	Submucosal
Trigone vs. Non-trigone	Both	Both	Both	Trigone	Non-trigone	Both
Distribution Local vs. Diffuse	Local	Diffuse	Diffuse	Local	Diffuse	Local
Number of injections	10	20	20	10	30	10
Dilution volume (mL)	0.5	0.5	1.0	0.5	1.0	1.0

* Patients at highest risk for incomplete bladder emptying (Diabetes Mellitus (DM), age > 70, Detrusor Hyperactivity with Impaired Contractile Function (DHIC), CIC averse).

**Table 3:** Proposed checklist for future Onabotulinum toxin A injection paradigm studies to ensure adequate reporting of study details.

Category	Details to Collect/Report
Methods	❏ Study Design (e.g., RCT, Prospective Cohort, Retrospective Cohort) ❏ Study Population ❏ Demographics (e.g., Age, Sex, Race/Ethnicity, Frailty status) ❏ OAB-wet vs. OAB-dry ❏ Other special populations ❏ Primary outcome ❏ Sample Size Calculation ❏ Follow up time
Procedure Details	❏ Anesthesia Details (choose 1 or more) ❏ Local anesthesia technique ❏ General Anesthesia ❏ Sedation details ❏ Dose (Units) ❏ Details of dilution strategy) (Units/mL) ❏ Anatomical location ❏ Trigone-inclusive ❏ Trigone sparing ❏ Number of injections ❏ Volume of injections ❏ Distribution of Injections ❏ Bladder base ❏ Bladder walls ❏ Both ❏ Spread of Injections ❏ Local ❏ Diffuse ❏ Procedure duration ❏ Needle gauge and length ❏ Scope type and sheath size
Outcomes	Therapy Success ❏ Subjective Outcomes ❏ Validated questionnaires (PROs e.g., PGI-I, TBS, OAB-q, ICIQ-SF, OABSS, other relevant tools) ❏ Objective Outcomes ❏ Urgency, Urgency Incontinence, Micturition Episodes (e.g., Bladder diary) ❏ UDS parameters ❏ Other outcomes: ❏ Pain/Tolerability (e.g., VAS, NPRS) ❏ Willingness to repeat therapy ❏ Time to next injection
Adverse Events	❏ Urinary retention requiring CIC ❏ UTI ❏ Hematuria

CIC: Clean Intermittent Catheterization, ICIQ-SF: International Consultation on Incontinence Questionnaire Short Form, NPRS: 11-point Numerical Pain Rating Scale, OABSS: Overactive Bladder Symptom Score, PGI-I: Patient Global Impression of Improvement, PROs: Patient Reported Outcomes, RCT: Randomized Controlled Trial, TBS: Treatment Benefit Scale, UDS: Urodynamic Study, UTI: Urinary Tract Infection. VAS: 10-point visual analog scale

## Data Availability

No new data were created or analyzed in this study.

## Supplementary Materials

The following supporting information can be downloaded at https://www.mdpi.com/article/10.3390/toxins17050211/s1. Table S1: Data Extraction Table; Table S2: Injection Volume, Number, Depth and Location.
