# Supplementary material for: Intravesical Onabotulinum Toxin A Injection Paradigms for Idiopathic Overactive Bladder: A Scoping Review of Clinical Outcomes, Techniques, and Implications for Practice and Future Research
Source: Toxins (Basel). 2025 Apr 23;17(5):211. doi: 10.3390/toxins17050211 (PMC12115849; doi:10.3390/toxins17050211)
Supplement: Supplementary file 1 [file toxins-17-00211-s001.zip › Supplemental Table S1. BTXA Injection Paradigm Data Extraction Table .pdf]

Study	Study title	Study design	Sample size (by group)	% Female	Mean/median age	Dose	Scope/needle	Anesthesia Type	Efficacy	Adverse events
Abdelwahab et al. 2015	Efficacy Of Botulinum Toxin Type A 100 Units Versus 200 Units For Treatment Of Refractory Idiopathic Overactive Bladder	**Randomized controlled trial** (2 groups: 100U vs 200U)	80<br>100U: 40<br>200U: 40	82.50%	**100U**: 30.2<br>**200U**: 31.4	100U, 200U	Rigid scope, 6F without side holes (Amecath Company®, Egypt) injection needle	Spinal anesthesia	**At 3 months, 100U vs 200U:**<br>Urgency episodes: -3.7 vs -3.22*<br>UUI: -1.02 vs -1.15 (p<0.05)*<br>Frequency: -1.2 vs -1.3 (p<0.05)*<br>Nocturia: - 0.74 vs - (p<0.05) *<br><br>Improved :clinical symptoms and UDS parameters<br><br>**At 3 months, QOL outcomes (100 vs 200U):**<br>OABSS score: - 6.6 vs -6.8 (p<0.05)*<br>HRQOL: 29.7 vs 36.5 (p<0.05)*<br><br>Significant improvement in OABSS and HRQOL at months 1, 3, 6 and 9 after treatment to baseline data in both groups (100U vs. 200U)<br><br>*change from baseline within treatment group P<0.05	**At 3 months, adverse events (100U vs 200U):**<br>UTI: 3/40 (7.5%) vs 7/40(17%)<br>Hematuria: 6/40 (15%) vs 9/40 (22.5%)<br>Urinary retention or CIC: Not reported
Altaweel et al. 2011	Prospective Randomized Trial Of 100U Vs 200U Botox In The Treatment Of Idiopathic Overactive Bladder	**Randomised controlled trial** (2 groups: 100U vs 200U)	22<br>100U: 11<br>200U: 11	NR	NR	100U, 200U	Rigid scope, 22F, 23g needle	IV general anesthesia	**At 3 months, 100U vs 200U:**<br>Urgency episodes: - 7.3 vs -6.4<br>UUI: -0.5 vs -0.1<br>Frequency: -7.8 vs -7.3<br>*Significant improvement in frequency, urgency and urinary incontinence compared to baseline, with no significant difference between the groups.*<br><br>**QOL was significantly improved in both groups with no difference between 100U vs. 200U**<br>UDI-6: -30 vs -30<br>IIQ-7: -30 vs -30	**At 3 months, adverse events (100U vs 200U):**<br>**UTI:** 1/11 (9%) vs 1/11 (9%)<br>**Hematuria:** 4/11 (36.3%) vs 5/11 (45.5%)<br>**Retention:** 1/11 (9.5%) vs 2/11 (18%)
Amundsen et al. 2016	OnabotulinumtoxinA vs Sacral Neuromodulation on Refractory Urgency Urinary Incontinence in Women: A Randomized Clinical Trial	**Randomised controlled trial** (2 groups: 200U vs SNM)	364<br>BTXA: 190<br>SNM: 174	100%	**BTXA**: 62.9<br>**SNM:** 63.1	200U	12 or 30-degree lens and rigid scope. 22 gauge	50 mL of 2% lidocaine placed in the bladder and 10 mL of 2% lidocaine jelly	**At 6 months (BTX vs SNM)**<br>UUI/day (mean change): - 3.9 vs - 3.25 (p<0.01)<br>Complete resolution UI: 20% vs 4% (p<0.001)<br>PGI-I: 71% vs 68% (p=0.82)<br>**At 6 months, Improvement in QOL**<br>Overactive Bladder Questionnaire SF: 41.6 vs 38.1 (p=0.17)<br>Symptom bother score: - 46.7 vs - 38.6 (p<0.002)	**At 6 months, adverse events:**<br>UTI: 35% vs 11% (p<0.001)<br>Elevated PVR (required CIC): 4/191 (2.1%) vs 0/178 (0%)
Brubaker et al. 2008	Refractory Idiopathic Urge Urinary Incontinence and Botulinum A Injection	**Multi-institutional, randomized, double-blind, placebo controlled trial** (2 groups: 200U vs Placebo)	43<br>BTXA: 28<br>Placebo: 15	100%	**BTXA**: 64.7<br>**Placebo**: 69.2	200U	Cystoscope	Local anesthesia	Approximately 60% in the BTXA group had a clinical response based on Patient Global Impression of Improvement (PGI-I). The median duration of the response was 373 days.<br><br>**At 2 months:** Mean PGI-I score after the initial injection was significantly better in the BTXA group (2.7 vs 4.0, p = 0.003).<br><br>**At one month:** there was a highly significant difference in the number of IEs and total IEs on a 3-day urinary diary (p <0.0001).<br><br>18/25 (72%) in the BTXA group who completed a second diary experienced more than a 75% decreased number of IEs. No subject in the control group experienced that level of reduction.<br><br>Patient perception of the adequacy of symptom control was significantly better in the BTXA group (p <0.0001).<br><br>Symptom bother was significantly improved in the BTXA group with a significant decrease in the UDI urge subscale score	**Elevaled PVR:** BTXA 12/28 (43%)<br>**Self catheterization:** BTXA 9/28 (32%)<br>**UTI:** BTXA (44%), Placebo (22%)
Chang et al. 2022	Postprocedural Pain Associated With 5 Versus 20 Intradetrusor Injections of OnabotulinumtoxinA for Treatment of Overactive Bladder: A Multicenter Randomized Clinical Trial	**Randomised controlled trial** (2 groups: 5 vs 20, detrusor, trigone sparing injections)	60<br>5 injection: 30<br>20 injection: 30	100%	64.1	100 U	23g, Laborie InjeTAK, or Coloplast BoNee needle, Rigid cystoscopy, 20F Sheath	local anesthesia	**GRA efficacy scores:(p=0.52)**<br>5 injection: Moderate improvement (+2; IQR: +1 to +3)<br>20 injection: Moderate improvmeent (+2; IQR: +1 to +3)<br><br>**Procedure time (Median time) (p<0.001)**<br>5 injection: 76 seconds (IQR: 58-127 seconds)<br>20 injection: 176 seconds (IQR; 106-224 seconds)<br><br>**VAS scores: (p=0.27)**<br>5 injection: 2 (IQR: 1-4)<br>20 injections: 3 (IQR 2-4)<br><br>Perceived pain, efficacy, and postprocedure complications did not significantly differ between patients receiving 5 injections and 20 injections.There was no difference in patient satisfaction, but the 5 injection procedure was significantly shorter	**UTI: (p=0.74)**<br>5 injection: 6/30: 20%<br>20 injection: 5/30; 16.7%<br><br>**CIC: (p=0.99)**<br>5 injection:3/30; 10%<br>20 injection: 6/30; 6.7%
Chapple et al. 2013	OnabotulinumtoxinA 100 U Significantly Improves All Idiopathic Overactive Bladder Symptoms and Quality of Life in Patients with Overactive Bladder and Urinary Incontinence: A Randomised, Double-Blind, Placebo-Controlled Trial	**Randomised controlled trial** (2 groups: 100U vs Placebo)	548<br>BTXA: 277<br>Placebo: 271	**BTXA:** 88.1% vs **Placebo:** 84.5%	**BTXA**: 59.5<br>**Placebo**: 59.2	100 U	Flexible or rigid scope	local anesthesia or sedations	**At week 12 (placebo vs BTXA):**<br>UI episodes: -16.8 vs -53.1% (p<0.001)<br>UUI episodes: -13.9 vs -53.2% (p<0.001)<br>Micturition episodes: -6 vs -19.7% (p<0.001)<br>Urgency episdoes: -8.4 vs -41.1% (p<0.001)<br>Nocturia: -8.8% vs -25.1% (p<0.01)<br>I-QOL: 6.3 vs 23.1 (p<0.001)<br><br>Decreased number of urgency, UI, UUI, and nocturia episodes per day at week 12 compared to placebo<br>Improved QOL	**At week 12 (placebo vs BTXA):**<br>UTI: 5.2% vs 20.4%<br>Hematuria: 0.4 vs 3.6%<br>Elevated PVR (>200): 1.1 vs 8.8%<br>CIC: 0.7 vs 6.9%%
Cohen et al. 2009	Preliminary Results of a Dose-Finding Study for Botulinum Toxin-A in Patients With Idiopathic Overactive Bladder: 100 Versus 150 Units	**Randomised controlled trial** (2 groups: 100U vs 150U)	44<br>100U: 20<br>150U: 24	NR	NR	100 U (n=20), 150 U (n=24)	14F flexible cystoscope, 27g Olympus needle, 4mm needle length	local anesthesia	**At 12 weeks, 100U vs 150U:**<br>Reduction in frequency episodes:<br>OAB-dry: - 6.1 vs -13.6 * (p=0.8)<br>*higher baseline severity<br><br>Reduction in UI episodes:<br>OAB wet: - 6 vs - 6.5 (p=0.33):<br><br>Improved QOL (measured via VAS):<br>No difference in QOL<br>100U vs. 150U:<br>VAS change: -5.6 (100U) vs -5.8 (150U) (p=NS)	**At 12 weeks, 100U vs 150U:**<br>UTI: 7/42 (16%)<br>Elevated PVR (>350mL, unablel to void) with CIC: 2/42 (4.5%)<br>1 in each group 100 and 150U
DiCarlo-Meacham et al. 2023	Reduced versus standard intradetrusor OnabotulinumtoxinA injections for treatment of overactive bladder	**Randomized noninferiority trial** (2 groups: 5 vs 20 detrusor, trigone sparing injections)	83<br>5 injection: 41<br>20 injection: 42	97.60%	59.8	100 U (control (20)n=42 and study (5)n=41)	NR	NR	The control group showed greater improvement in OAB-q QOL scores than the study group (31.8 vs. 21.3; p = 0.04), though no significant differences were found in OAB-q SB or ICIQ-SF scores.<br><br>The reduced injection technique did not meet noninferiority criteria compared to the control. Both groups experienced pain post-procedure (p < 0.001), but pain changes were similar (p = 0.94).<br><br>More participants in the reduced injection group were willing to repeat the procedure (OR = 3.8; p = 0.004)	**Adverse events:**<br>UTI: 6/41(14.6%) vs 11/42 (26.2%)<br>CIC: 2/41 (4.9%) vs 0/42 (0%)<br>Hematuria: 0/42 (0%) vs 1/42 (2.4%)
Dmochowski et al. 2010	Efficacy and Safety of OnabotulinumtoxinA for Idiopathic Overactive Bladder: A Double-Blind, Placebo-Controlled, Randomised, Dose-Ranging Trial	**Randomised controlled trial** (6 groups: 50U vs 100U vs 150U vs 200U vs 300Uvs placebo)	313<br>Placebo: 43<br>50U: 56<br>100U: 55<br>150U: 50<br>200U: 52<br>300U: 55	92%	58.8	50, 100, 150, 200 or 300 U	Flexible or rigid scope	local anesthesia or sedations	Among the 212 patients with baseline DO who had data available for this analysis, 33% (70/212) did not have DO recorded at week 12:<br>**DO Resolution:**<br>Placebo 7/31 (22.5%)<br>50U 15/43(34.9%)<br>100U 11/36 (30.6%)<br>150U 10/31 (32.3%)<br>200U 16/36 (44.4%)<br>300U 11/35 (31.4%)<br><br>**UUI Resolution (incontinence free):**<br>Placebo 15.9%<br>50U 29.8%<br>100U 37.0%<br>150U 40.8%<br>200U 50.9%<br>300U 57.1%	Dose-dependent adverse events observed included rates of urinary tract infection (UTI) and urinary retention (post residual volume > 200mL) requiring clean intermittent catheterization (CIC).<br><br>**Retention with CIC rates:**<br>Placebo: 0%<br>50 U dose: 5.4%<br>100U: 10.9%<br>**150U: 20%**<br>200U: 21.2%<br>300U: 16.4%<br><br>**UTI rates:**<br>Placebo: 16.3%<br>50U: 33.9%<br>100U: 36.4%<br>150U: 44%<br>200U: 48.1%,<br>300U: 34.5%

El-Azab et al. 2013	The Satisfaction Of Patients With Refractory Idiopathic Overactive Bladder With Onabotulinumtoxina And Augmentation Cystoplasty	**Non-randomized Prospective Cohort study** (2 groups: 100U or 200U BTXA vs Augment Cystoplasty (AC)) (by patient preference)	31<br>BTXA: 16<br>AC: 15	**BTXA**: 43.8%<br>**AC**: 73%	**BTXA**: 24.5<br>**AC**: 28.2	100U or 200U	5 mm, 23 g needle, on a 5-F sheath; Contigen Injection Needle, introduced through a 22-F rigid cystoscope	NR	**At 3 months: BTXA vs AC**<br>Frequency: -1.7 vs -2.17<br>UI: -1.54 vs -2.39<br>SUI: -0.4 vs -0.8<br><br>There were significant improvements in urinary symptoms (UDI-6) and quality of life (IIQ-7) after both procedures	**At 3 months:**<br>CIC: BTXA 2/16 (12.5%) vs. AC 4/15 (26.6%)
El-Hefnawy et al. 2021	Trigonal-Sparing Versus Trigonal-Involved Botox Injection For Treatment Of Idiopathic Overactive Bladder: A Randomized Clinical Trial	**Randomised controlled trial** (2 groups: 20 Trigone sparing vs 20 Trigone inclusive injections)	103<br>Trigone involved: 51<br>Trigone-sparing: 52	Overall: 80% (82/103)<br>**Trigone involved**: 82.4%<br>**Trigone sparing**: 76.9%	**Trigone involved**: 34.3 ± 10<br>**Trigone-sparing**: 33.3 ± 10	100U	rigid cystoscope ,7g needle	Sedation (propofol and ketamine)	Clinical outcome were (trigone sparing vs trigone inclusive):<br>UI episodes (1 months): −93% and −85% (p=0.18)<br>UI episodes (3 months): −77% and −87% (p=0.38)	**At 3 months, trigone involved vs trigone sparing**<br>**UTI:** 6 [11.7%] vs 5 [9.6%],respectively; (p = 0.343)<br>**Subjective voiding difficulties:** 10/51 (19.6%) versus 3/52 (5.7%) (P = 0.052)<br>**CIC (with PVR >200mL):** 2/51 (3.9%) vs 0/52 (0%)
Flynn et al. 2009	Outcome of a Randomized, Double-Blind, Placebo Controlled Trial<br>of Botulinum A Toxin for Refractory Overactive Bladder	**Randomised controlled trial** (2 groups: Placebo vs 200/300U BTXA)	22<br>BTXA: 15<br>Placebo: 7	100%	66	200/300U (pooled analysis of both doses)	22 g needle, 14Fr rigid cystoscope	2-stage study, data for 200 and 300U were combined	**At 6 weeks, clinical outcomes**<br>**Incontinence episodes** -57.5 vs 9.3% (p<0.01).<br>**IIQ-7**: -67.3 vs 0% (p<0.01)<br>**UDI-6:** -37.5 vs 7.4% (p<0.02)<br>**24 hr pad weight:** -45 vs -2.3% (p 0.02)<br>**Void per day:** -12.2 vs -6.8% (p = 0.08)	**UTI:** BTXA: 13% vs placebo: 28%<br>**Elevated (PVR >200U):** BTXA: 26.5% vs placebo: NR<br>**CIC:** BTXA: 1/22 (4.5%) vs placebo: NR
Fowler et al. 2012	OnabotulinumtoxinA Improves Health-Related Quality of Life in Patients With Urinary Incontinence Due to Idiopathic Overactive Bladder: A 36-Week, Double-Blind, Placebo-Controlled,<br>Randomized, Dose-Ranging Trial.	**Randomised controlled trial: 3 month follow up study** (6 groups: 50U vs 100U vs 150U vs 200U vs 300Uvs placebo)	313<br>Placebo: 44<br>50U: 57<br>100U: 54<br>150U: 49<br>200U: 53<br>300U: 56	288/313 (92%)	**50U:** 58.2 **100U:** 60.8 **150U:** 56.9 **200U:** 59.6 **300U:** 58.7	50U, 100U, 150U, 200U, or 300U	Flexible or rigid scope	local anesthesia or sedations	At 36 weeks, QOL outcomes (primary outcomes):<br>Treatment with doses of 100-300U decreased symptom burden and improved disease-specific quality of life and general HRQOL in patients with idiopathic OAB and UUI whose symptoms were inadequately managed by oral antimuscarinics. Importantly, similar benefit was observed for doses 100- 300 U in many of the HRQOL measures used, with diminishing incremental gain for doses >150 U. Clinically meaningful benefits observed among treatment groups as early as 2 week after treatment and were sustained for up to 30–36 weeks.	NR
Ginsberg et al. 2017	Long-Term Treatment With Onabotulinumtoxina Results In Consistent, Durable Improvements In Health-Related Quality Of Life In Patients With Overactive Bladder.	**Randomised controlled trial**: 3 year extension trial (follow up study of pooled phase 3 trials) (Single arm: 100U)	829	90.30%	60.1	100U	Flexible or rigid scope	local anesthesia or sedations	Improvements in QOL (I-QOL and KHQ)<br>Most patient achieved or exceeed the MID for IQOL and KHQ scores. 72.9% achieved or exceeded teh MID for IQOL after treatment and all subsequent treatements (1-6 treatments)	UTI: most commonly reported AE (no details)<br>CIC: 4% after treatent 1, and <2% aftrer subsequent treatments
Granese et al. 2012	Botox For Idiopathic Overactive Bladder: Efficacy, Duration<br>And Safety. Effectiveness Of Subsequent Injection	**Prospective Cohort study** (Single arm: 100U)	68	100%	56	100U	rigid scope, flexible needs, 18g, 10 mm	general anesthesia	**At 3 month, change in clinical outcomes from baseline (on 4 day voiding diary):**<br>Urgency episodes/day: -7.8<br>UI episodes/day: - 3.9<br>Micturitions/day: -9.9<br><br>Reduction in daily urgency episodes and daily frequency episodes. Significant improvement in urodynamic parameters, clinical features and quality of life, after the first injection of BTX until the 9 months of follow-up	**Elevated PVR (>100mL):**<br>At 1 month: 24/68 (35%)<br>At 2 months: 15/68 (22%)<br>At 3 months: 2/68 (3%)<br><br>**Elevated PVR + LUTS requiring CIC:**<br>At 1 month: 8/68 (11.7%)<br>At 2 months: 5/68 (7.4%)<br>At 3 months: 1/68 (1.5%)<br><br>UTI: NR
Herschorn et al. 2017	The Efficacy and Safety of OnabotulinumtoxinA or Solifenacin Compared with Placebo in Solifenacin Naïve Patients with Refractory Overactive Bladder: Results from a Multicenter, Randomized, Double-Blind Phase 3b Trial	**Multicenter, Randomized, Double-Blind Phase 3b Trial** (3 groups: BTXA 100U vs Solifenacin vs Placebo)	356<br>BTXA: 145<br>Solifenacin: 151<br>Placebo: 60	308/356 (86.5%)	62	100U	NR	NR	**Outcomes at three months (100U BTX vs. placebo):**<br><br>Daily incontinence episodes: -3.19 vs. 1.33 (p <0.001)<br><br>Daily micturition episodes (95% CI): -2.3 (-2.7, -1.9) vs. -1.1 (-1.7, -0.4) (p= 0.002)<br><br>Daily nocturia episodes (95% CI): -0.6 (-0.7, -0.4) vs. -0.3 (-0.5, 0.0) (p= 0.088)<br>100% continent: 33.8% vs. 11.7%<br>Number of positive TBS response: 97/136 (71.3%) vs. 26/58 (44.8%) (p <0.001)	**Adverse events at any point in treatment cycle (100U BTX vs. placebo)**<br>UTI: 37/145 (25.5%) vs. 6/60 (10.0%)<br>Urinary retention: 10/145 (6.9%) vs. 0
Hsiao et al. 2016	Factors Associated with Therapeutic Efficacy of Intravesical OnabotulinumtoxinA Injection for Overactive Bladder Syndrome	**Prospective Cohort study** (Single arm: 100U)	89	48.30%	64.7	100U	NR	NR	**At 3 months after treatment, significant clinical improvements were observed (p<0.001):**<br>Urgency episodes/3 days: - 4.4<br>UUI episodes/3 days: - 2.7<br>Frequency episodes/3 days: - 3.4<br>Functional Bladder capacity:<br>Resolution of OAB-wet: 62.9%<br><br>**Improved QOL:**<br>OABSS score: -2.6<br><br>*change from baseline within treatment group P<0.05	NR
Karakeçi et al. 2019	Efficacy And Safety Of Botulinum Neurotoxin In Geriatric Patients With An Overactive Bladder: A Multicentric Study From Turkey	**Retrospective Cohort study** (Single arm: 100U)	34	50%	72.3	100U	20F rigid cystoscope		**At 3 months, staticallly signicant improvements were observed in clinical outcomes (p<0.05):**<br>Urinary frequency/day: -5.7<br>Urinary incontinence/day: -2.6<br>Nocturia: -2.1<br>Pad use/day: -1.5<br><br>*At 3 and 6 months post treatment, authors observed a statistically significant decrease in the number of episodes of urinary frequency, nocturia, daily pad usage and number of incontinence episodes.*<br><br>**At 3 months, improved QOL scores:**<br>I- QOL score: 25.2<br>TBS (improved): 70.6%<br><br>Improved QOL (I-QOL)<br>Patient reported improvement and patient satisifcation (TBS)	**At 3 months, adverse events:**<br>UTI: 5/34 (14.7%)<br>Hematuria: 3/34 (8.8%)<br>Elevated PVR requiring CIC: 6/32 (17.7%)
Karsenty et al. 2007	Botulinum Toxin Type A Injections Into The Trigone To Treat Idiopathic Overactive Bladder Do Not Induce Vesicoureteral Reflux.	**Prospective cohort study** (Single arm: 200U)	11	100%	76	200U	Rigid cystoscope (Stortz injection system), disposable, flexible endoscopic needle (23 G)	Local anesthesia	**Outcomes at 6 weeks (N=11)**<br><br>1/9 baseline incontinent patients reported dry at 6 weeks<br><br>2/9 baseline incontinent patients had >50% reduction in the number of incontinence episodes/ day at 6 weeks<br><br>3/4 patients with baseline detrusor overactivity resolved it at six weeks<br><br>4/11 reported improvement that made them ask for another injection<br><br>Median difference in V8 OAB Questionnaire score: -6.0 (P= 0.019)	**Adverse events:** Pain related to injection was reported by patients with a mean of 4/10
Kuo et al. 2007	Comparison of Effectiveness of Detrusor, Suburothelial and Bladder Base Injections of Botulinum Toxin A for Idiopathic Detrusor Overactivity.	**Randomised controlled trial** (3 groups: 40 detrusor, trigone sparing; 40 suburothelial trigone sparing; 10 suburothelial, bladder base)	45<br>Detrusor:15<br>Suburothelial: 15<br>Bladder base: 15	38% (17/45)	**Suburothelial/ Bladder wall:**<br>72.1 ±10.3<br>**Detrusor/Bladder wall**<br>71.6 ± 13.6<br>**Suburothelial/ Bladder base:**<br>67.9 ± 12.1	100U	NR	NR	**At 3 months, there were no statistically significant differences in efficacy.**<br>Success Rates (3, 6, 9 months) p = 0.025<br>Detrusor wall injections: 93%. 67%. 20%<br>Suburothelial bladder wall: 80%. 47%. 20%<br>Suburothelial bladder base: 67% 13%. 6.7%<br><br>Bladder body (detrusor + suburothelial) vs Bladder base: p= 0.01<br><br>Dry rates (Resolution of DO/Urinary continence): p = 0.7<br>Detrusor wall: 60%<br>Suburothelial bladder wall: 47%<br>Suburothelial bladder base: 53%	CIC were lower in the trigone inclusive bladder base group (0% vs 13.3%).<br>No VUR was observed.

Kuo, H. C. 2011	Bladder Base/Trigone Injection Is Safe And As Effective As Bladder Body Injection Of Onabotulinumtoxina For Idiopathic Detrusor Overactivity Refractory To Antimuscarinics.	**Single blind, randomized, paralleled, actively controlled trial** (3 groups: Bladder body vs Bladder body/trigone vs Bladder base/trigone)	105<br>Bladder body: 37<br>Bladder body/trigone: 35<br>Bladder base/trigone: 33	54%	**Bladder body:** 65.1<br>**Bladder body/trigone:** 66.5<br>**Bladder base/trigone**: 68.3	100U	Rigid cystoscopic injection (22 Fr, Richard-Wolf) 23-gauge needle	General anesthesia	**Outcomes at 3 months**, (3 months vs baseline) Bladder body vs Bladder body/trigone vs Bladder base/trigone:<br><br>Mean difference in frequency: -12.6 vs -12.6 vs -14.6 (P= 0.9)<br>Mean difference in urgency: 0.4 vs -3.86 vs -2.96 (P= 0.83)<br>Mean difference in UUI: -9.23 vs -7.62 vs -6.24 (P= 0.93)<br>Mean difference in urgency and UUI: -8.83 vs -11.5 vs -9.20 (P=0.91)<br>Dry rates: 73% vs 65.7% vs 78.8%<br><br>Success rates (3, 6, 9, 12 months):<br>Bladde body: 71% 56% 49%. 49%<br>Bladder body/trigone: 74% 50%. 50%. 50%<br>Bladder base/Trigone: 73%. 72%. 44%. 37%	**Adverse events:** Bladder body vs Bladder body/trigone vs Bladder base/trigone:<br><br>UTI: 8/37 (21.6%) vs 9/35 (25.7%) vs 5/33 (15.2%); (P= 0.56)<br><br>Hematuria: 6/37 (16.2%) vs 4/35 (11.4%) vs 3/33 (9.1%) (P=0.833)<br><br>Large PVR: 16/37 (43.2%) vs 13/35 (48.5%) vs 16/33 (48.5%) (P=0.639)
Kuschel et al. 2008	Botulinum Toxin-A For Idiopathic Overactivity Of The Vesical Detrusor: A 2-Year Follow-Up.	**Prospective cohort study** (single arm: 100U)	26	100%	66	100U	rigid cystoscope	spinal or general anesthesia	**Objective QOL outcomes**<br>11 patients of the single injection group<br>80% of patients reported improved Household activities<br>70% improved Outdoor activities<br>40% improved ability to travel<br>20% effects on nocturnal sleep<br>20% wearing pads	UTI: 1/26 (3.9%)<br>Elevated PVR (>100mL): 2/26 (7.7%)
Liao et al. 2016	Different Number of Intravesical OnabotulinumtoxinAInjections for Patients With Refractory Detrusor Overactivity Do Not Affect Treatment Outcome:A Prospective Randomized Comparative Study	**Randomized Controlled trial** (3 groups: 10 vs 20 vs 40 suburothelial, trigone sparing injections)	67<br>10 injection: 24<br>20 injection: 22<br>40 injection: 21	49%	65	100U	rigid 22F cystoscope, 23 g, 1mm	light intravenous general anesthesia	**At 6 months, clinical improvements were observed (10 vs 40 vs 20):**<br>UUI episodes: 7.24 vs 4.53 vs 0.15. UTI rates were higher in the 20-injection group (31.8%) compared to the 10- (12.5%) and 40-injection groups (9.5%), with no significant differences in other adverse events. Pain scores or duration of procedures were not assessed.<br><br>Both the 10- and 40-injection groups demonstrated significant reductions in UUI but not the 20 injection group.<br><br>**QOL outcomes:**<br>no significant differences were found in OAB-q SB or ICIQ-SF scores. The reduced injection technique did not meet noninferiority criteria	UTI rates were higher in the 20-injection group (31.8%) compared to the 10- (12.5%) and 40-injection groups (9.5%), with no significant differences in other adverse events.<br>Pain scores or duration of procedures were not assessed.<br><br>Urinary retention:<br>10 injections: 3 (12.5%)<br>20 injections: 1 (4.5%)<br>40 injections: 2 (9.5%)<br><br>PVR >200 ml:<br>10 injections: 41.7%<br>20 injectinos: 59.1%<br>40 injections: 38.1%
MacDiarmid et al. 2024	Efficacy And Safety Of An Alternative Onabotulinumtoxina Injection Paradigm For Refractory Overactive Bladder	**Randomized controlled trial** (2 groups: 100U vs placebo)	120<br>BTXA: 80<br>Placebo: 40	**BTXA** : 75/80 (93.8%)<br>**Placebo:** 40/40 (100%)	**BTXA:** 60.5<br>**Placebo:** 61.9	100 U	Rigid or flexible cystoscope	local anesthesia	The BTXA group demonstrated significantly greater reductions in at week 12<br><br>Daily UI episodes: (−2.9) versus placebo (−0.3) (least squares mean difference [LSMD]: −2.99, p < 0.0001).<br><br>Improvements in QOL (I-QOL and KHQ)<br>Most patient achieved or exceeed the MID for IQOL and KHQ scores. 72.9% achieved or exceeded teh MID for IQOL after treatment and all subsequent treatements (1-6 treatments)	**At 12 weeks, adverse events:**<br>UTI: BTXA 12/78 (15.4%) vs Placebo 2/39 (5.1%)<br>Retention (required CIC in 2 patients): BTXA 2/78 (2.6%) vs. Placebo 0/39 (0%)<br>CIC: BTXA 2/78 (2.6%) vs. Placebo 0/39 (0%)<br>Dysuria: BTXA 4/78 (5.1%) vs. Placebo 1/39 (2.6%)<br>VUR was not reported.
McCammon et al. 2021	Early and Consistent Improvements in Urinary Symptoms and Quality of Life With OnabotulinumtoxinA in Patients With Overactive Bladder and Urinary Incontinence: Results From a Randomized, Placebo-controlled, Phase IV Clinical Trial	**Randomized controlled trial** (2 groups: 100U vs Placebo)	254<br>BTXA: 129<br>Placebo: 125	89%	60.8	100U	Rigid or flexible cystoscope	local anesthesia	**At 3 months, statistically signifcant improvments in clinical outomes from baseline (p<0.05) (placebo vs BTXA 100U):**<br>UI episodes/day: -1.6 vs 3.5<br>Dry rates: 7.2% vs 32.0%<br><br>**Improved QOL (KHQ):**<br>KHQ: - 13.5 vs -34.4	**At 3 months:, adverse events:**<br>UTI: 8/125 (6.4%) vs 27/128 (21.1%)<br>Elevated PVR with CIC: 0/125 (0%) vs 8/128 (6.3%)
Mohanty et al. 2008	Role of botulinum toxin-A in the management of refractory idiopathic detrusor overactive bladder: Single-centre experience	**Prospective Cohort study** (arm: 100U)	39	100%	52	200U	Rigid Cystoscopy, 23g Cook, 5 mm	Regional anesthesia	**At 3 months, improvement in clinical outomes from baseline within 1 week of injection:**<br>Urgency: approx -3 (e.g. 15-16 (baseline) to 6-7 (3 months)<br>UI: approx -2 (e.g. 4-5 (baseline) to 2-3 (3 months)<br>Frequency: approx - 9 (e.g. 15-16 (baseline) to 6-7 (3 months)<br><br>*Improvement observed within 1 week of injection, which lasted for mean period of 7 months*<br><br>Improved UDS parameters (including volume at first desire to void improved from median baseline of 104-204 ml and maximum cystometric capacity of bladder increased from mean baseline value of 205-330 ml)	None<br>- No retention or UTIs
Mühlstädt et al. 2018	Quo Vadis Botulinum Toxin: Normative Constraints and Quality of Life for Patients With Idiopathic OAB?	**Retrospective observational study** (Single arm: 100U)	51	76.50%	63.5	100 U	Rigid endoscope	general anesthesia	Decreased frequency (decreased from 10.4 ± 0.5 to 5.2 ± 0.4 micturitions per day)<br>Decreased number of pads used (3.6 ± 1.0 to 1.2 ± 0.3 pads per day)<br>Improved QOL and patient satisifcation (CSQ-8 and KHQ)	NR
Nitti et al. 2013	OnabotulinumtoxinA for the Treatment of Patients with Overactive Bladder and Urinary Incontinence: Results of a Phase 3, Randomized, Placebo Controlled Trial.	**Randomized controlled trial** (2 groups: 100U vs Placebo)	254<br>BTXA: 280<br>Placebo: 277	**BTXA**: 90%<br>**Placebo**: 88.4%	**BTXA**: 61.7<br>**Placebo**: 61	100 U	Flexible or rigid scope, 2mm	local anesthesia or sedations	**At 3 months:**<br>UI episodes: BTXA –2.65 vs Placebo –0.87, (P<0.001)<br>Urgency: BTXA –1.21 vs Placebo –2.93, (P<0.001)<br>Nocturia: BTXA –0.24 vs Placebo –0.45, (P<0.05)<br><br>**UUI resolution (p<0.001)**<br>50% resolution:<br>Placebo: 28.9%<br>100U: 57.5%<br><br>100% resolution:<br>Placebo: 6.5%<br>100U: 28.9%<br><br>**Positive treatment response at 12 weeks:**<br>Placebo: 29.2%<br>100U: 60.8%<br><br>**Mean change I-QOL from baseline:**<br>BTXA 21.9 vs Placebo 6.8, (P<0.001)<br>Mean change KHQ from baseline: improved in BTXA group	**At 3 months:**<br><br>UTI : BTXA 43/ 278 (15.5%) vs placebo 16/272 (5.9%)<br>Retention: 15/278 (5.4%)<br>PVR >200: BTXA 24/278 (8.7%) vs Placebo 0/278<br>CIC: BTXA 17/278 (6.1%) vs Placebo 0/278
Nitti et al. 2016	Durable Efficacy and Safety of Long-Term OnabotulinumtoxinA Treatment in Patients with Overactive Bladder Syndrome: Final Results of a 3.5-Year Study.	**Randomised controlled trial** (2 groups: 100U vs 150U)	829<br>100U: 543<br>150U: 286	90.30%	60.1	100 U, 150 U	Flexible or rigid scope	local anesthesia or sedations	Urgency (100U group): -3.8<br>UI: -3.3<br>Micturition: - 2.6<br>No noted difference in efficacy between 100U and 150U<br><br>Reductions in episodes of urinary incontinence<br>Improvement in QOL (I-QOL): : 26<br>TBS: 76% with positive TBS score	**At 3 months:**<br>UTI: 141/829 (17 %)<br>Dysuria: 46/829 (5.5%)<br>Urinary retention PVR> 200- 350 ml : 32/829 (3.9%)<br>CIC due to retention: 34/ 829 (4.1%)
Okamura et al. 2013	Twelve Months Follow-up of Injection of OnabotulinumtoxinA into Vesical Submucosa for Refractory Non-neurogenic Overactive Bladder	**Prospective cohort study** (Single arm: 100U)	17	47%	67	100 U	NR	General or regional anesthesia	UUI, urgency and daytime urination significantly decreased up to the 11th month<br>Improved QOL and patient satisification (OABSS and ICIQ-UI SF)	PVR > 200 mL 2/17 (11.7%)
Onem et al. 2018	Efficacy and safety of onabotulinumtoxinA injection in patients with refractory overactive bladder: First multicentric study in Turkish population	**Cohort study** (Single arm: 100U)	80	60%	47.4	100U	Rigid or flexible cystoscopy 4 mm	Local, general, or sedation anesthesia	**At 3 months clinical outomes from baseline (p<0.05)**<br>Urinary frequency: -6.4 (P < 0.05)<br>UI episodes: -5.8 (P < 0.05)<br>Urgency episodes: -6.8 (P < 0.05)<br>Mean bladder capacity, and maximal bladder capacity were increased<br><br><br>I-QOL: 28.3 point improvement<br>Patient satisification and patient reported improvement (TBS): 82.5%	**At 3 months:**<br><br>UTI: 5/80 (6.25%)<br>Hematuria: 5/80 (6.25%)<br>High PVR volume and retention: 3/80 (3.8%)<br>CIC: 3/80 (3.8%)
Ospina-Galeano et al. 2018	Use of onabotulinum toxin A in patients with idiopathic overactive bladder and a lack of efficacy, intolerance or contraindication with anticholinergics.	**Prospective, cohort study** (Single arm: 100U/200U)	73	100%	58.9	100U, 200U	NR	NR	**At follow up, clinical improvements were observed:**<br><br>Decrease in UUI episodes (56% reduction)<br>Decresed in urgency episodes (41% reduction)<br>Decrease in daytime frequency (48% reduction)<br>Decrease in nighttime frequency (50% reduction)<br>Decrease in number of pads used (82%)<br><br>Improved UDS parameters (the first desire to void volume, cystometric capacity and volume of first involuntary detrusor contraction)<br>No QOL was not ultimately measured/analyzed	UTI: 1/73 (1.4%)<br>Urinary retention with CIC: 5/73 (6.8%)<br>No hematuria

Ou et al. 2023	Intravesical Injection of Botulinum Toxin Type A in Patients with Refractory Overactive Bladder—Results between Young and Elderly Populations, and Factors Associated with Unfavorable Outcomes	**Retrospective cohort review** (2 groups Young group vs Elderly group)	192<br>Young group:127<br>Elderly group: 65	**Young group**: 65.4%<br>**Elderly group**: 24.5%	**Young group**: 58.8<br>**Elderly group**: 82.0	100U	NR	NR	**Elderly (≥75) vs Young (<75) group**<br><br>**Subjective success rates:**<br>At 3, 6, 12 months: (p=NS)<br>Younger 97.6%, 77.2%, 24.4%<br>Elderly: 96.9%, 84.6%, 32.3%<br><br>**Subjective cure (dry) (p=0.34)**<br>Younger: 66.9%<br>Elderly: 60%	**Elderly (≥75) vs Young (<75) group**<br><br>**UTI (p=0.33)**<br>Young: 14.2%<br>Elderly: 9.2%<br><br>**Retention (requiring CIC): (p=0.42))**<br>Young: 8.7%<br>Elderly: 12.3%<br><br>*Adverse event rates, including urinary retention, elevated PVR, and UTI, did not differ between groups
Owen et al. 2017	Comparison of the effectiveness of repeated injections of onabotulinum toxin A for refractory idiopathic detrusor overactivity: analysis of an open label extension of a randomized ttrial (the RELAX study)	**Randomised controlled trial** (2 groups: 200U BTXA vs Placebo)	240<br>BTXA: 122<br>Placebo: 118	100%	NR	200U	NR	NR	**Average patient symptoms at 6 weeks following each repeated BTX injection**<br><br>Incontinence episodes/day:<br>1 Injection (n=201): 2.53 (3.76)<br>2 Injections (n=133): 2.35 (3.85) (p 0.14)<br>3 Injections (n=50): 1.71 (3.44) (p 0.08)<br><br>Urgency episodes/day:<br>1 Injection (n=197): 3.74 (4.15)<br>2 Injections (n=136): 3.28 (3.92) (p 0.66)<br>3 Injections (n=50): 3.34 (4.02) (p 0.49)<br><br>Voiding episodes/day:<br>1 Injection (n=204): 8.41 (3.39)<br>2 Injections (n=138): 8.24 (3.43) (p 0.27)<br>3 Injections (n=50): 7.99 (2.91) (p 0.11)	**Adverse events 6 weeks following each repeated BTX injection:**<br><br>UTI:<br>1 Injection: 48/228 (21%)<br>2 Injections: 61/155 (39%)<br>3 Injections: 20/59 (34%)<br><br>Difficulty voiding:<br>1 Injection: 47/228 (21%)<br>2 Injections 52/155 (34%)<br>3 Injections: 18/59 (31%)<br><br>There was no statistically significant difference between<br>the number of individuals experiencing UTIs or voiding<br>difficulty for each of the active injections.<br><br>Due to the number of missing data, these estimates should be interpreted with a degree of caution.
Rovner et al. 2011	Urodynamic Results and Clinical Outcomes With Intradetrusor Injections of OnabotulinumtoxinA in a Randomized, Placebo-Controlled Dose-Finding Study in Idiopathic Overactive Bladder	**Randomised controlled trial** (6 groups: 50U vs 100U vs 150U vs 200U vs 300Uvs placebo)	313<br>Placebo: 44<br>50U: 57<br>100U: 54<br>150U: 49<br>200U: 53<br>300U: 56	92%	58.8	50U, 100U, 150U, 200U, or 300U	flexible or rigid cystoscope (as per local practice)	local anesthesia (with or without sedation as per local practice)<br><br>9.3% of all patients received sedatives in addition to the local anes thesia	**At 3 Months:** Weekly Frequency of OAB Symptoms Mean Changes From Baseline at 3 Months, According to Presence or Absence of Detrusor Overactivity (DO) at Baseline:<br><br>Number of UUI episodes:<br>Patients with DO, change at week 12:<br>Placebo: -17.7<br>50U: -21.1 (P < 0.05)<br>100U: -18.6<br>150U: -24 (P < 0.001)<br>200U: -20 (P < 0.05)<br>300U: -19.7 (P < 0.01)<br>Patients without DO, change at week 12:<br>Placebo: -16.3<br>50U: -19.2<br>100U: -17.6<br>150U: -20.9<br>200U: -18.2<br>300U: -18.1	**At 3 months**, adverse events:<br>UTI:<br>Placebo: 7/43 (16.3%)<br>50U: 19/56 (33.9%)<br>100U: 20/55 (36.4%)<br>150U: 22/50 (44%)<br>200U: 25/52 (48.1%)<br>300U: 19/55 (34.5%)<br><br>Urinary Retention:<br>Placebo: 1/43 (2.3%)<br>50U: 5/56 (8.9%)<br>100U: 10/55 (18.2%)<br>150U: 14/50 (28.0%)<br>200U: 12/52 (23.1%)<br>300U: 14/55 (25.5%)<br>(P <0.05 vs. placebo for all groups ≥100 U)
Schulte-Baukloh et al. 2022	Real-Time Documentation of the Effect of Onabotulinumtoxin A Detrusor Injection in OAB Patients—Preliminary Results	**Pre-post intervention study** (Single arm: 100U)	17	88% (15/17)	64.6	100U	rigid cystoscope (17 or 21 French)	local anesthesia or general anesthesia depending on patient preferences	**At day 20, clinical outcomes:**<br><br>Mean frequency/24 hours:<br>5.75 (SD: 0.50) episodes/day at day 20 compared to 12.83 (SD: 5.54) episodes/day at baseline (p <0.001)<br><br>Daytime frequency:<br>4.25 (SD: 0.96) daytime episodes of frequency at day 20 compared to<br>10.87 (SD: 5.16) daytime episodes at baseline (p= 0.001)<br><br>Nightime frequency<br>1.50 (SD: 1.29) nightime episodes of frequency at day 20 compared to 1.96 (SD: 0.98) nightime episodes of frequency at baseline (p= 0.049)	UTI: 2/17 (12%)
Sievert et al. 2014	Onabotulinumtoxina 100U Provides Significant Improvements In Overactive Bladder Symptoms In Patients With Urinary Incontinence, Regardless Of The Number Of Anticholinergic Therapies Used Or The Reason For Inadequate Management Of Overactive Bladder	**Randomised controlled trial** (2 groups: 100U vs Placebo)	1105<br>BTXA: 557<br>Placebo: 548	87.8% (970/1105)	Overall: 60.4<br><br>**Placebo**: 60.1<br>**BTXA 100U**: 60.6	100U	NR	NR	**At week 12, 100U vs Placebo:**<br>Daily UI episodes: -2.80 vs. -0.95 episodes/day; p < 0.001<br><br>Daily episodes of urgency: -3.30 vs. -1.23;p < 0.001<br><br>Daily micturition episodes -2.35 vs. -0.87; p < 0.001<br><br>≥ 50% reduction from baseline in UI episodes: 60.5% vs. 31.0%; p < 0.001<br><br>100% reduction in UI episodes: 27.1% vs. 8.4%;p < 0.001	**Adverse events at any point in treatment cycle (100U BTX vs. placebo):**<br>UTI: 141/552 (25.5%) vs. 52/542 (9.6%)<br>Hematuria: 18/552 (3.3%) vs. 18/542 (3.3%)<br>Urinary Retention: 32/552 (5.8%) vs. 2/542 (0.42%)
Singh et al. 2015	Comparison of Short Term Outcomes of Sacral Nerve Stimulation and Intradetrusor Injection of OnabotulinumtoxinA (Botox) in Women With Refractory Overactive Bladder	**Retrospective cohort study** (2 groups: SNS vs BTXA (100U vs 200U vs 300U)	128 total<br>BTXA: 63<br>SNS: 65	100%	**SNM**: 64.5 (13.62)<br>**BTXA:** 66.7 (11.5)	100U, 200U, 300U	NR	NR	Main outcome: Treatment failure, defined as less than 50% improvement from baseline symptoms or retreatment for OAB symptoms within 6 months of the initial BTX injection.<br><br>**At six months post-treatment:**<br>Treatment success overall was 68.2% (43/63).<br>Success at six months post-treatment for each individual dose was 100U: 93.3% (14/15)<br>200U: 56.4% (22/39)<br>300U: 77.8% (7/9)	**At six months, adverse events:**<br>UTI: 31/63 (49%)<br>Urinary retention requiring clean intermittent self-catheterization: 22/63 (35%)
Tamburro et al. 2018	Onabotulinumtoxin-A Improves Health Status And Urinary Symptoms In Subjects With Refractory Overactive Bladder: Real-Life Experience	**Prospective study** (Single arm: 100U/150U)	22	77%	62.8	100U, 150U	rigid cystoscope	Regional anesthesia	**At 12 weeks, significant reductions were observed:**<br>Urinary Incontinence: -2.6 (p<0.001)<br>Frequency: -5.5 (p<0.001)<br>Nocturia: -2 (p<0.001)<br>Pads/3 days: -1.69 (p<0.001)<br>Improvement in QOL:<br>36-Item Short-Form Health Survey: significant improvement in several domains<br>Overactive Bladder Screener: -16.9 (p<0.05)<br>Patient reported improvement and patient satisfaction (TBS)::<br>Greatlly improved or improved (87%)	**At 12 weeks, adverse events:**<br>Retention (CIC): 2/22 (9%)
Ton et al. 2021	Outcomes Of A Single Trigone-Only Vs. 20 Trigone-Sparing Injections Of Onabotulinumtoxina For Refractory Overactive Bladder (OAB)	**Retrospective Chart Review** (2 groups: 1 suburothelial, trigone only vs 20 detrusor trigone sparing injections)	45<br>Trigone only: 19<br>Trigone-sparing: 26	NR	**Trigone only:** 68 (10)<br>**Trigone-sparing:** 68 (13.2)	100U	Rigid scope	NR	Outcomes measured did not include changes in OAB symptoms such as episodes of urgency, UI, frequency, or nocturia. The authors compared trigone-only and trigone-sparing BTX injections and measured outcomes which included inter-injection interval.<br><br>**Mean inter-injection interval (days):**<br>Trigone-only (1x10mL inj) vs. Trigone-sparing (20x0.5mL inj): 177 ± 72.0 (59–638) vs. 168 ± 55.1 (91–498) (p= 0.373)<br>**Procedure time:** lower in the single injection group (4.3 ± 2.02, 5.7 ± 2.9, p =0.003)	**Adverse events trigone-only (1x10mL injection) vs. trigone-sparing (20x0.5mL injections)**<br>UTI: 15.4% ± 36.4 vs. 17.6 ± 38.2 (p= 0.703)<br>Urinary retention with catheterization: 5.3% ± 22.4 vs. 17.4% ± 38.1 (p= 0.014)<br><br>Differences in pain scores were not assessed or reported
Visco et al. 2012	Anticholinergic Therapy vs. OnabotulinumtoxinA for Urgency Urinary Incontinence	**Double-blind, double-placebo–controlled, randomized trial** (2 groups: Anticholinergic Drug vs 100U)	472<br>BTXA: 121<br>Anticholinergic Drug: 126	100%	**BTXA:** 59.3<br>**ACH:** 56.7	100U	NR	NR	**Outcomes at six months (100U BTX vs. anticholinergic):**<br><br>Mean reduction in episodes of UUI/day:<br>3.3 vs. 3.4 (p= 0.81)<br><br>Complete resolution of UUI:<br>30/112 (27%) vs. 16/119 (13%) (p= 0.003)<br><br>Complete resolution of all incontinence:<br>26/112 (23%) vs. 13/119 (11%) (p= 0.003)<br><br>Change from baseline in score on OABq-SF, Symptom-severity scale: −44.08 vs. −44.55 (p= 0.87)<br><br>Change from baseline in score on OABq-SF, QOL scale:<br>37.13 vs. 37.05 (p= 0.98)	**Adverse events at six months (100U BTX vs. anticholinergic):**<br>Any adverse event: 88/120 (73%) vs. 88/127 (69%) (p= 0.79)<br>Intermittent catheterization: 1/106 (1%) vs. 0 (p= 0.49)<br>Self-catheterization since previous visit: 5/111 (5%) vs. 1/116 (1%) (p= 0.10)
