# Supplementary material for: Intravesical Onabotulinum Toxin A Injection Paradigms for Idiopathic Overactive Bladder: A Scoping Review of Clinical Outcomes, Techniques, and Implications for Practice and Future Research
Source: Toxins (Basel). 2025 Apr 23;17(5):211. doi: 10.3390/toxins17050211 (PMC12115849; doi:10.3390/toxins17050211)
Supplement: Supplementary file 1 [file toxins-17-00211-s001.zip › Supplemental Table S2. Injection Volume, Number, Depth and Location .pdf]

Study	Study design	Dose	Injection Volume	Number of injections	Concentration	**Injection Depth:** Detrusor vs suburothelial	**Injection Location:** Trigone sparing (Bladder body/walls only) vs Trigone inclusive	Key findings	Adverse events
Chang et al. 2022	**Randomised controlled trial** (2 groups: 5 vs 20, detrusor, trigone sparing injections)	100U	2mL (5 injections) vs 0.5 mL (20 injections)	5 vs 20 injection	100U/10mL	detrusor (3-4mm)	Trigone sparing	Perceived pain, efficacy, and postprocedure complications did not significantly differ between patients receiving 5 injections and 20 injections, but procedure time was significantly shorter	NR
DiCarlo-Meacham et al. 2023	**Randomized controlled trial** (2 groups: 5 vs 20 detrusor, trigone sparing injections)	100U	2mL (5 injections) vs 0.5mL (20 injections)	5 vs 20 injection	100U/10 mL	detrusor	Trigone sparing	The 20 injection group showed greater improvement in OAB-q QOL scores than the study group (31.8 vs. 21.3; p = 0.04), though no significant differences were found in OAB-q SB or ICIQ-SF scores.<br><br>The reduced injection technique did not meet noninferiority criteria compared to the control. Both groups experienced pain post-procedure (p < 0.0001), but pain changes were similar (p = 0.94).<br><br>More participants in the reduced injection group were willing to repeat the procedure (OR = 3.8; p = 0.004)	**Adverse events:**<br>UTI: 6/41(14.6%) vs 11/42 (26.2%)<br>CIC: 2/41 (4.9%) vs 0/42 (0%)<br>Hematuria: 0/42 (0%) vs 1/42 (2.4%)
El-Hefnawy et al. 2021	**Randomized controlled tria**l (2 groups: 20 Trigone sparing vs 20 Trigone inclusive injections	100U	0.5 mL	20 injections	100U/10mL	detrusor	Trigone sparing vs trigone inclusive	Clinical outcome were (trigone sparing vs trigone inclusive):<br>UI episodes (1 months): −93% and −85% (p=0.18)<br>UI episodes (3 months): −77% and −87% (p=0.38)	**At 3 months, trigone involved vs trigone sparing**<br>**UTI:** 6 [11.7%] vs 5 [9.6%],respectively; (p = 0.343)<br>**Subjective voiding difficulties:** 10/51 (19.6%) versus 3/52 (5.7%) (P = 0.052)<br>**CIC (with PVR >200mL):** 2/51(3.9%) vs 0/52 (0%)
Karsenty et al. 2007	**Prospective cohort study** (Single group: 200U)	200U	1 mL (10 injections)	10 injections	200U/10mL	detrusor	Trigone inclusive	**Outcomes at 6 weeks (N=11)**<br><br>1/9 baseline incontinent patients reported dry at 6 weeks<br><br>2/9 baseline incontinent patients had >50% reduction in the number of incontinence episodes/ day at 6 weeks<br><br>3/4 patients with baseline detrusor overactivity resolved it at six weeks<br><br>4/11 reported improvement that made them ask for another injection<br><br>**Median difference in V8 OAB Questionnaire score: -6.0 (P= 0.019)**	**Adverse events:** Pain related to injection was reported by patients with a mean of 4/10
Kuo et al., 2007	**Randomised controlled trial** (3 groups: 40 detrusor, trigone sparing; 40 suburothelial trigone sparing; 10 suburothelial, bladder base)	100U	0.5 mL	10 vs 40 injections	100U/20mL vs 100U/5mL	detrusor vs suburothelial	Trigone sparing vs trigone inclusive* bladder base	**At 3 months, there were no statistically significant differences in efficacy.**<br>Success Rates (3, 6, 9 months) p = 0.025<br>Detrusor wall injections: 93%. 67%. 20%<br>Suburothelial bladder wall: 80%. 47%. 20%<br>Suburothelial bladder base: 67% 13%. 6.7%<br><br>Bladder body (detrusor + suburothelial) vs Bladder base: p= 0.01<br><br>Resolution of DO/Urinary continence: p = 0.7<br>Detrusor wall: 60%<br>Suburothelial bladder wall: 47%<br>Suburothelial bladder base: 53% of patients<br><br>QOL improvements: p = 0.562<br>Detrusor wall: 93%<br>Suburothelail bladder wall: 80%<br>Suburothelial bladder base 87%<br><br>Success rate (general satisfaction rating of “excellent” or “moderately improved”)	CIC were lower in the trigone inclusive bladder base group (0% vs 13.3%).<br>No VUR was observed.
Kuo, H. C. 2011	**Single blind, randomized, paralleled, actively controlled trial**<br>(3 groups: Bladder body vs Bladder body/trigone vs Bladder base/trigone)	100 U	0.5mL (20 injections) vs 1mL (10 injections)	10 vs 20 injection	100U/10mL	suburothelial	Trigone sparing vs trigone inclusive bladder body vs trigone inclusive bladder base	"Outcomes at 3 months, (3 months vs baseline)<br>Bladder body vs Bladder body/trigone vs Bladder base/trigone:<br><br>Mean difference in frequency: -12.6 vs -12.6 vs -14.6 (P= 0.871)<br><br>Mean differenc in urgency: 0.4 vs -3.86 vs -2.96 (P= 0.827)<br><br>Mean difference in UUI: -9.23 vs -7.62 vs -6.24 (P= 0.927)<br><br>Mean differene in urgency and UUI: -8.83 vs -11.5 vs -9.20 (P=0. 905)<br><br>Dry rates: 73% vs 65.7% vs 78.8%<br>Success rates: 71% vs 74% vs 73%	**Adverse events:** Bladder body vs Bladder body/trigone vs Bladder base/trigone:<br><br>UTI: 8/37 (21.6%) vs 9/35 (25.7%) vs 5/33 (15.2%); (P= 0.56)<br><br>Hematuria: 6/37 (16.2%) vs 4/35 (11.4%) vs 3/33 (9.1%) (P=0.833)<br><br>Large PVR: 16/37 (43.2%) vs 13/35 (48.5%) vs 16/33 (48.5%) (P=0.639)
Kuschel et al. 2008	**Prospective cohort study**	100U	1 mL	30 injections	100U/30mL	detrusor	Trigone sparing	Day- time frequency after BTXA (9.5 ± 3.0 vs 11.4 ± 2.9)<br>Nocturia were reduced (1.9 ± 0.5 vs 2.1 ± 1) compared to before BTX-A injection<br><br>Objective QOL outcomes<br>11 patients of the single injection group<br>80% of patients reported improved Household activities<br>70% improved Outdoor activities<br>40% improved ability to travel<br>20% effects on nocturnal sleep<br>20% necessity of wearing pads	UTI: 1/26 (3.9%)<br>Elevated PVR (>100mL): 2/26 (7.7%)

Liao et al. 2016	**Randomized controlled trial** (3 groups: 10 vs 20 vs 40 suburothelial, trigone sparing injections)	100U	1.0mL (10 injections) Vs 0.5 mL (20 injections) Vs 0.25 mL (40 injections)	10 vs 20 vs 40 injections	100U/10mL	suburothelail (1 mm)	Trigone sparing	**At 6 months, clinical improvements were observed (10 vs 40 vs 20):**<br>UUI episodes: 7.24 vs 4.53 vs 0.15. UTI rates were higher in the 20-injection group (31.8%) compared to the 10- (12.5%) and 40-injection groups (9.5%), with no significant differences in other adverse events. Pain scores or duration of procedures were not assessed.<br><br>Both the 10- and 40-injection groups demonstrated significant reductions in UUIm but not the 20 injection grou.<br><br>**QOL outcomes:**<br>no significant differences were found in OAB-q SB or ICIQ-SF scores. The reduced injection technique did not meet noninferiority criteria	UTI rates were higher in the 20-injection group (31.8%) compared to the 10- (12.5%) and 40-injection groups (9.5%), with no significant differences in other adverse events.<br>Pain scores or duration of procedures were not assessed.<br><br>Urinary retention:<br>10 injections: 3 (12.5%)<br>20 injections: 1 (4.5%)<br>40 injections: 2 (9.5%)<br><br>PVR >200 ml:<br>10 injections: 41.7%<br>20 injectinos: 59.1%<br>40 injections: 38.1%<br><br>UTI:<br>10 injections: 12.5 %<br>20 injections: 31.8%<br>40 injections: 9.5%<br><br>There were no significant differences in the rates of hematuria, dysuria, urinary retention, bladder pain, micturition pain, a large PVR volume, and UTI between the groups
McDiarmid et al. 2024	**Randomized controlled trial** (2 groups: 100U vs placebo)	100U	0.5 mL	10 injections	100U/5mL	detrusor (2mm)	Trigone inclusive* bladder base	The BTXA group demonstrated significantly greater reductions in<br>at week 12<br><br>Daily UI episodes: (−2.9) versus placebo (−0.3) (least squares mean difference [LSMD]: −2.99, p < 0.0001).<br><br>Improvements in QOL (I-QOL and KHQ)<br>Most patient achieved or exceeed the MID for IQOL and KHQ scores. 72.9% achieved or exceeded teh MID for IQOL after treatment and all subsequent treatements (1-6 treatments)	**At 12 weeks, adverse events:**<br>UTI: BTXA 12/78 (15.4%) vs Placebo 2/39 (5.1%)<br>Retention (required CIC in 2 patients): BTXA 2/78 (2.6%) vs. Placebo 0/39 (0%)<br>CIC: BTXA 2/78 (2.6%) vs. Placebo 0/39 (0%)<br>Dysuria: BTXA 4/78 (5.1%) vs. Placebo 1/39 (2.6%)<br>VUR was not reported.
Okamura et al. 2013	**Prospective cohort study** (1 group: 30 suburothelial, trigone sparing injections)	100U	0.5 mL	30 injections	100U/15mL	suburothelial	Trigone sparing	UUI, urgency and daytime urination significantly decreased up to the 11th month<br>Improved QOL and patient satisification (OABSS and ICIQ-UI SF)	PVR > 200 mL 2/17 (11.7%)
Onem et al. 2018	**Prospective cohort study** (1 group: 20 detrusor, trigone inclusive injections)	100U	1 mL	20 injections	100U/20mL	detrusor (4mm)	Trigone inclusive*	**At 3 months clinical outomes from baseline (p<0.05)**<br>Urinary frequency: -6.4 (P < 0.05)<br>UI episodes: -5.8 (P < 0.05)<br>Urgency episodes: -6.8 (P < 0.05)<br>Mean bladder capacity, and maximal bladder capacity were increased<br><br>I-QOL: 28.3 pt improvement<br>Patient satisification and patient reported improvement (TBS): 82.5%	**At 3 months:**<br><br>UTI: 5/80 (6.25%)<br>Hematuria: 5/80 (6.25%)<br>High PVR volume and retention: 3/80 (3.8%)<br>CIC: 3/80 (3.8%)
Ton et al. 2021	**Retrospective cohort study** (2 groups: 1 suburothelial, trigone only vs 20 detrusor trigone sparing injections)	100U	10 mL (1 injection) vs 0.5mL (20 injections)	1 vs 20 injections	100U/10mL	suburothelial vs detrusor	Trigone sparing vs Trigone inclusive	Outcomes measured did not include changes in OAB symptoms such as episodes of urgency, UI, frequency, or nocturia. The authors compared trigone-only and trigone-sparing BTX injections and measured outcomes which included inter-injection interval.<br><br>**Mean inter-injection interval (days):**<br>Trigone-only (1x10mL inj) vs. Trigone-sparing (20x0.5mL inj):<br>177 ± 72.0 (59–638) vs. 168 ± 55.1 (91–498) (p= 0.373)<br><br>**Procedure time:** lower in the single injection group (4.3 ± 2.02, 5.7 ± 2.9, p =0.003)	**Adverse events trigone-only (1x10mL injection) vs. trigone-sparing (20x0.5mL injections)**<br>UTI: 15.4% ± 36.4 vs. 17.6 ± 38.2 (p= 0.703)<br>Urinary retention with catheterization: 5.3% ± 22.4 vs. 17.4% ± 38.1 (p= 0.014)<br><br>Differences in pain scores were not assessed or reported
Zdroik et al. 2024	**Randomized controlled trial** (2 groups: 10 vs 20, detrusor, trigone sparing injections)	100U	1 mL (10 injections) vs 0.5mL (20 injections)	10 vs 20 injection	100U/10mL	detrusor	Trigone sparing	**Outcomes at 3 months, 20 injection vs 10 injections:**<br><br>Difference in median pain score: 3 [1–4] vs 4 [1.5–5] , p=0.82<br><br>Success: 40% (2/5) vs 46.7% (7/15) (p= 0.795)<br><br>Voids per 24 hours: 7.00 (4.84–8.33) vs. 7.00 (6.00–8.33) (p= 0.62)<br><br>Leaks per 24 hours: 1.35 (0–2.00) vs. 1.64 (1–1.82) (p= 0.55)<br><br>Pad changes per 24 hrs: 1.00 (0–2.50) vs. 0.67 (0–2.00) (p= 0.94)<br><br>Success defined as achieving 50% or greater reduction in the number of UUI episodes at 12 weeks post-injection	Adverse events at 2 weeks<br>10 injections vs 20 injections<br>Overall AE, p=0.84<br>UTI: 2/21 (9.5%) vs 2/19 (10.5%)<br>Hematuria: 1/21 (4.8%) vs. 0/19 (0%)<br>CIC: 1/21 (4.8%) vs. 2/19 (10.5%)"
